# Enzymatic Encoding of Topology in an Intrinsically Disordered Single‐Chain Protein

**DOI:** 10.1002/anie.3738676

**Published:** 2026-05-26

**Authors:** Joshua Johani, Kristin Eichelberger, Olga Guskova, Simbulele Charlotte Dunjwa, Hans Bolinsson, Anna‐Maria Börjesdotter, Lars Nilsson, Doris Jaros, Harald Rohm, Albena Lederer

**Affiliations:** ^1^ Leibniz‐Institut Für Polymerforschung Dresden e.V. Dresden Germany; ^2^ Department of Chemistry and Polymer Science Stellenbosch University, Private Bag X1 Stellenbosch South Africa; ^3^ Chair of Food Engineering Institute of Natural Materials Technology Technische Universität Dresden Dresden Germany; ^4^ CoSAXS Beamline, MAX IV Laboratory and Department of Process and Life Science Engineering Faculty of Engineering LTH Lund University Lund Sweden

**Keywords:** enzymatic cross‐linking, intrinsically disordered proteins, SEC‐SAXS, single‐chain nanoparticles, topology encoding

## Abstract

Controlling the three‐dimensional topology of single‐chain nanoparticles (SCNPs) remains a central challenge in polymer and protein chemistry, particularly for intrinsically disordered systems lacking defined secondary structure. Here, we demonstrate that selective enzymatic intramolecular cross‐linking can encode topologically biased interactions in an intrinsically disordered protein (IDP), yielding compact SCNPs with reproducible cavity architecture. Using β‐casein‐rich sodium caseinate (βNaCn) as a model surrogate for bovine β‐casein (β‐Cn), microbial transglutaminase (mTGase) introduces sparse, sequence‐resolved glutamine‐lysine isopeptide bonds that drive reproducible chain collapse without inducing secondary structure. Analyses by size exclusion chromatography with quintuple detection (SEC‐D5), cross‐linking mass spectrometry (XL‐MS), molecular dynamics (MD) simulations, and SEC coupled to synchrotron small‐angle x‐ray scattering (SEC‐SAXS) converge to reveal a topology combining a stable, compact, hydrophobic core with flexible, disordered loops. These cavities are probed using Nile red (NR) fluorescence and SEC‐SAXS, which together provide topology information via guest‐induced density redistribution after NR capture. This work establishes that sparse enzymatic constraint installation, combined with residue‐resolved cross‐link mapping and orthogonal structural analysis, can encode and validate topology in a disordered single chain, thereby placing IDP‐like covalent folding in direct conceptual continuity with SCNP design.

## Graphical abstract

Enzymatic intramolecular cross‐linking enables topology control in intrinsically disordered proteins, yielding SCNPs with reproducible cavity architectures. Using β‐casein and microbial transglutaminase, sparse, sequence‐resolved constraints drive compact collapse without inducing secondary structure. XL‐MS, SEC‐SAXS and MD reveal a hydrophobic core with flexible loops and guest‐responsive internal density.

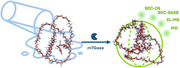

## 1 Introduction

Encoding three‐dimensional topology into single‐chain macromolecules in solution is a central unsolved problem at the intersection of polymer chemistry, physics and biomolecular folding. Single‐chain nanoparticles (SCNPs) generally are linear polymers that can collapse into dense nanoparticles by intrachain cross‐linking, driven by covalent or non‐covalent interactions [1, 2]. They provide a conceptual route to protein‐like compaction within a single chain [2, 3], yet most synthetic SCNPs remain trapped between Gaussian coils and dense globules, yielding heterogeneous topologies whose internal architecture is difficult to predict or validate [4]. In parallel, protein‐derived nanoparticles are often assembled from multiple chains or higher‐order aggregates, obscuring the mechanistic link between intrachain connectivity and emergent topology [5, 6]. A decisive advance would be a strategy that (i) installs a small number of long‐range constraints at defined residue positions, (ii) preserves local disorder (i.e., does not invoke stable secondary or tertiary structure), and (iii) enables topology to be validated by orthogonal, quantitative analysis.

Intrinsically disordered proteins (IDPs) offer an unusually stringent testing ground. Their conformational ensembles are broad, yet their sequences encode non‐random patterns of charge, hydrophobicity, and flexibility that control intrachain contacts and responsiveness to sparse constraints [7, 8, 9, 10]. Prior single‐chain collapse studies have shown that compact nanoparticles can be generated from synthetic macromolecules by introducing long‐range cross‐links via ring‐closing metathesis reaction [11], varying chain lengths and ligation density [12], and, more recently, from proteins such as denatured BSA by chemical intramolecular cross‐linking with ester‐forming linkers of varying spacer lengths [13]. In biological settings, sparse covalent modifications and binding events can reshape IDP ensembles dramatically. To translate this principle into chemical design, the question stretches beyond mere chain collapse to whether sparse, residue‐level constraints can be installed under mild conditions into a genuinely disordered single chain and whether the resulting topology can be validated experimentally. Enzymes are uniquely positioned for this purpose. Compared with small‐molecule cross‐linkers, enzymes provide substrate selectivity and operate under aqueous, near‐physiological conditions [14, 15]. We therefore test the hypothesis that site‐selective enzymatic “constraint installation” can encode reproducible topology in a disordered single chain. In this context “constraint installation” means that while the exact positions of cross‐links are not pre‐programmed, the experimentally identified long‐range constraint hotspots are reproducible, sufficient to confirm structural compaction, and demonstrate that sparse residue‐level connectivity edits are sufficient to encode topology.

## 2 Results and Discussion

**FIGURE 1:**
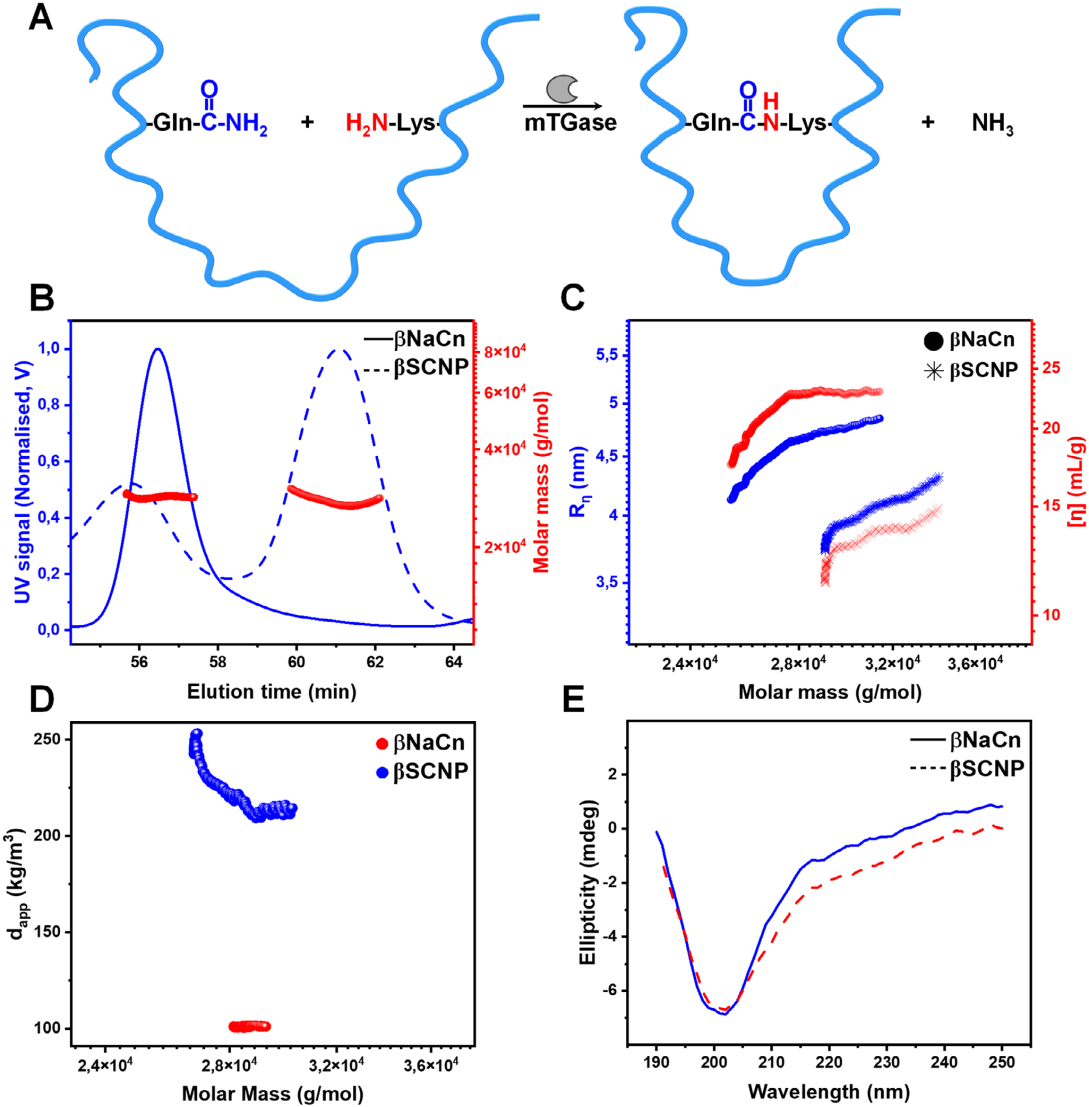
Enzymatic constraint installation quantified by orthogonal contraction analysis (SEC‐D5). (A) Concept: microbial transglutaminase (mTGase) induces sparse ε‐(γ‐glutamyl)lysine isopeptide bonds into a single β‐casein chain eventually leading to a compact single‐chain nanoparticle (βSCNP). (B) SEC‐D5 chromatograms (βNaCn vs βSCNP) show a forward retention‐time shift consistent with reduced hydrodynamic volume while maintaining essentially constant molar mass. (C) Hydrodynamic parameters (R_η_, [η]) quantify contraction at constant M_w_. (D) Apparent density d_app_ increases upon folding. (E) Circular dichroism shows no emergence of new secondary structure upon compaction. Contraction parameters and definitions are given in Supporting Information (Equations S7–S13).

As a model disordered substrate, we use β‐casein‐rich sodium caseinate (βNaCn), a practical surrogate for bovine β‐casein (β‐Cn; UniProt P02666) [16]. β‐Cn is intrinsically disordered, lacks disulfide bonds, and contains a high density of reactive glutamine/lysine residues (approx. 14% combined), which are distributed heterogeneously along the chain [16, 17]. As the enzyme we use microbial transglutaminase (mTGase; EC 2.3.2.13), which catalyses ε‐(γ‐glutamyl)‐lysine isopeptide formation between accessible Gln/Lys residues [15, 18]. βNaCn was dissolved at low concentration (0.5 g kg^−1^) to obtain IDP chains in the monomeric state [19], and incubated with 3 U mTGase g^−1^ protein at 40°C for up to 24 h (Figure 1A; full experimental details in Supporting Information). These dilute conditions were chosen to minimise intermolecular self‐association and thereby favour unimolecular collapse during cross‐linking. Unlike conventional protein stabilisation or gelation [20, 21], this system lacks a preexisting fold, but its compaction emerges from sparse, enzyme‐installed long‐range intrachain constraints, placing the resulting casein single‐chain nanoparticles in direct conceptual continuity with synthetic SCNPs.

Discriminating intramolecular collapse from multichain association remains a central challenge in translating SCNP concepts to disordered macromolecules. Common polymer characterisation techniques are often inadequate for characterisation of amphiphilic IDP‐like substrates, including casein SCNPs, as noncovalent interactions are strongly influenced by solvent, pressure, concentration and temperature [22, 23]. We therefore quantified SCNP compaction applying an orthogonal topology analysis starting with SEC‐D5 [24] under denaturing conditions, where urea denatures proteins and prevents aggregation by disrupting non‐covalent, hydrogen bonds and increasing solvation of hydrophobic groups [25]. The high urea concentration is cumbersome in that increased electron density diminishes the contrast of sample‐to‐buffer by elevating the background noise in refractive index, viscosity and light scattering detection, but is imperative to obtain βNaCn in the monomeric form. Under these conditions, uncross‐linked βNaCn exhibits a narrow monomeric distribution, and mTGase treatment yields single‐chain nanoparticle βSCNP with a pronounced forward retention‐time shift consistent with reduced hydrodynamic volume while leaving molar mass effectively unchanged (Figure 1B). Specifically, βNaCn shows molar mass *M*_w_ = 27.2 kg mol^−1^ with viscosity radius *R*_η,w_ = 4.8 nm and intrinsic viscosity [η]_w_ = 24.96 mL g^−1^, whereas enzymatically cross‐linked βSCNP retains *M*_w_ ≈ 27.8 kg mol^−1^ with 25% and 56% reductions to *R*_η,w_ = 3.6 nm and [η]_w_ = 10.94 mL g^−1^, respectively (Table S3). While the unchanged molar mass after enzymatic cross‐linking confirms unimolecular collapse, the reduction in R_η_ and especially in [η] is a robust indicator of macromolecular structural changes (conformation, shape, branching) due to its sensitivity to physical and topological interactions of macromolecular chains with solvent molecules [26].

The constant molar mass with substantial decrease in size *R*_η_ (Figure 1C) explains a twofold increase in apparent macromolecular density (d_app_) of βSCNP (Figure 1D). Importantly, circular dichroism spectra (Figure 1E) confirm that βSCNP compaction retains the same random coil structure as βNaCn, exhibiting strong negative ellipticity at ∼200 nm with no characteristic minima at 222 nm (α‐helix) or 218 nm (β‐sheet). This is the defining experimental signature of SCNP formation, which directly supports the central premise that mTGase installs sparse intrachain Gln–Lys constraints rather than building oligomers or ordered secondary structure motifs [27]. To evaluate the collapse with quantitative physicochemical parameters, we describe βSCNP contraction relative to βNaCn using the scaling descriptors ν and α relating chain size and intrinsic viscosity to molar mass from the scaling law and the Mark–Houwink relation, respectively, and the contraction factors g and g′ which are based on the ratios of the SCNP‐precursor hydrodynamic size and intrinsic viscosity as a function of molar mass, respectively; the full derivations are provided in Equations S9–S13. Expectedly, due to the absence of a molar mass distribution in monodisperse proteins, the scaling parameters (ν, α) could not be determined reliably [12]. The resulting contraction factors for βSCNP: g ≈ 0.60, g′ ≈ 0.46, indicate substantial chain compaction [12, 28]. Although these values are consistent with synthetic SCNPs under theta‐like solvent conditions, the degree of compaction is likely overestimated due to the extended unfolding of the highly flexible βNaCn under denaturing conditions. Regardless, the contraction demonstrates that sparse constraint networks, rather than dense hydrogen‐bonding networks, can encode topology [29, 30].

**FIGURE 2:**
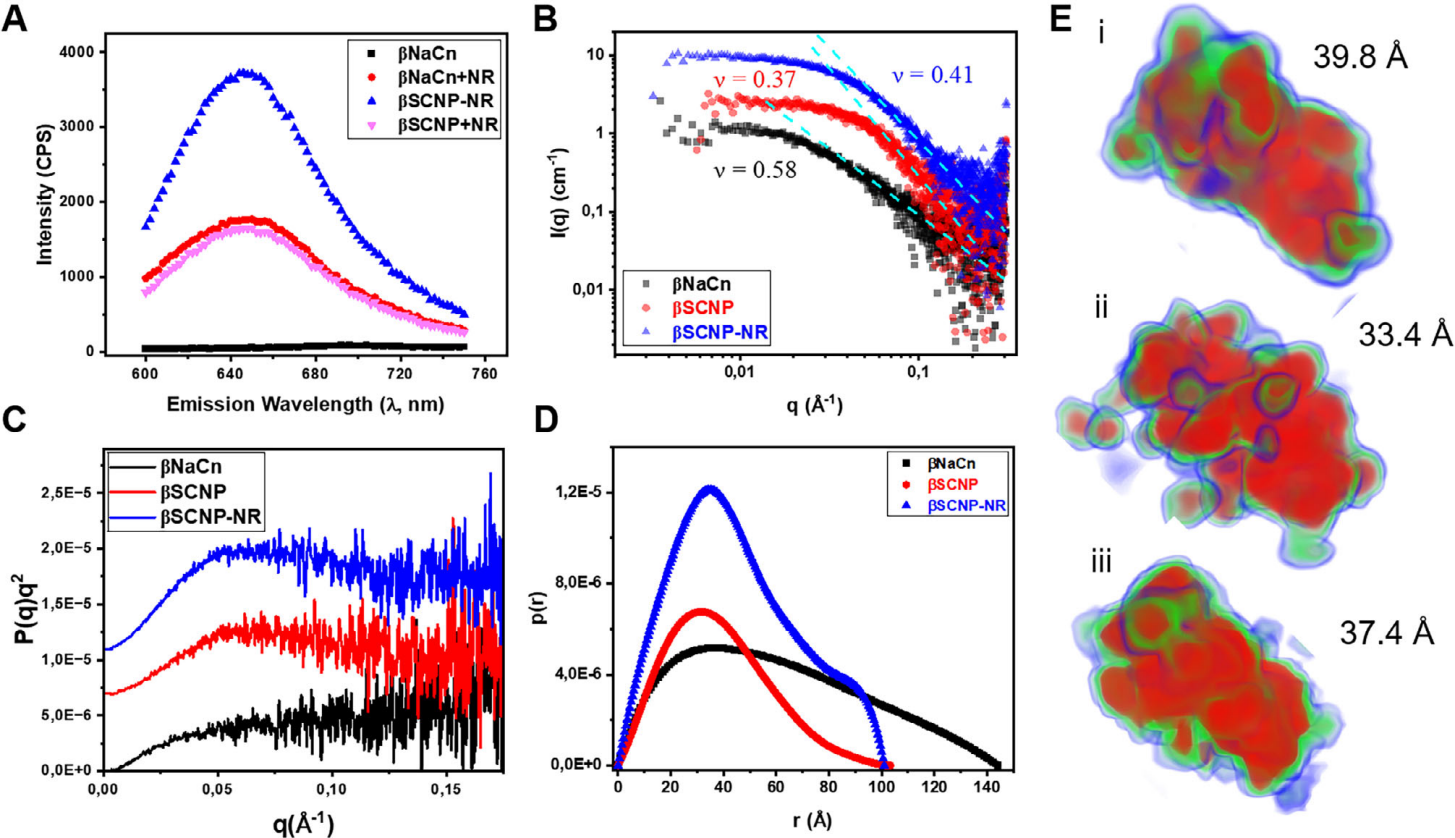
Nile red (NR) fluorescence and SEC‐SAXS prove topology impact. (A) NR fluorescence reports formation of hydrophobic microenvironments upon constraint‐driven folding. The signal is highest when NR is present during mTGase folding (βSCNP‐NR), consistent with guest capture during formation of the constrained ensemble rather than post‐adsorption. (B) Scattering profiles from the monomeric elution peak maxima of βNaCn, βSCNP, and βSCNP‐NR from SEC‐SAXS (see Figure S10), showing effective ν estimates obtained by analysing the intermediate‐q scattering regime (see Equation S22). (C) Kratky representations indicate increased compactness upon folding and altered internal packing upon NR capture. (D) Pair‐distance distribution functions P(r) show compaction for βSCNP and density redistribution for βSCNP‐NR. Structural parameters are summarised in Table 1. (E) Ab initio low‐resolution real‐space reconstructions from experimental solution scattering data of (i) βNaCn, (ii) βSCNP and (iii) βSCNP‐NR shown as volumes coloured according to electron density from low‐high in the order blue‐green‐red, and the estimated resolutions are shown next to each sample.

To test whether the enzymatically encoded fold produces topologically distinct internal compartments rather than a featureless collapsed globule, we employed Nile red (NR) as a solvatochromic indicator of hydrophobic void formation and connectivity [31, 32]. NR fluorescence rises strongly upon transfer from water into nonpolar microenvironments; thus, increased signal reports the emergence of sequestering pockets upon collapse. Figure 2A shows that βSCNPs display markedly higher NR fluorescence than βNaCn. The strongest response is obtained when cross‐linking is performed in the presence of NR (βSCNP‐NR), consistent with guest capture during formation of the folded topology rather than weak post‐adsorption at the surface.

To link this information to structural evidence, we then perform SEC‐SAXS on the monomeric elution peak. The SEC separation before synchrotron SAXS‐detection is essential here because it minimises polydispersity and interparticle effects, which otherwise smears SAXS interpretation in soft, disordered systems [33, 34]. Scattering curves and Kratky plots show that βNaCn behaves as a highly flexible chain, whereas βSCNP and βSCNP‐NR are markedly more compact (Figure 2B,C) [12, 29, 35]. The SAXS‐derived radius of gyration decreases from βNaCn (*R*_g_ = 5.3 ± 0.5 nm) to βSCNP (*R*_g_ = 3.0 ± 0.2 nm) and increases modestly for βSCNP‐NR (*R*_g_ = 3.8 ± 0.1 nm), while remaining far below the extended precursor (Table 1). These values are reproduced across independent determinations (Guinier, P(r), and DAMMIF) and model based molecular form factor analysis (Table 1). The SAXS‐derived scaling factor ν is an effective solution‐state scaling descriptor obtained from the Porod region of the scattering curve under the present SEC‐SAXS conditions, rather than a universal sequence‐intrinsic exponent. It provides the conformational basis for the changes in *R*_g_. For βNaCn, ν = 0.58 is consistent with a swollen random‐coil‐like conformation in good solvent, likely reflecting denaturation, whereas βSCNP and βSCNP‐NR show lower values (ν = 0.37–0.41) consistent with more compact conformations. Importantly, Kratky and P(r) analyses indicate that NR capture does not simply “swell” a soft particle; rather it redistributes internal density [36]. The βSCNP‐NR data exhibit a more pronounced compactness signature and a bimodal P(r), consistent with a constrained structure that supports partially shielded compartments whose internal packing is altered by guest occupancy (Figure 2C,D). Together with Figure 2E(i–iii), the ab initio low‐resolution reconstructions from SEC‐SAXS data provide an intuitive real‐space representation of the particles, facilitating comparison of their overall dimensions and density organisation, especially where high resolution crystal structures are unknown, as in this case [37, 38]. The reconstructions indicate that βSCNP‐NR differs from βNaCn and βSCNP in overall compactness and density distribution, in accordance with the scattering‐derived structural parameters. Under this interpretation NR plays the role of a topology probe, demonstrating that only structures that generate partially shielded pockets and constrained exchange pathways yield both the fluorescence enhancement and SAXS‐detectable density redistribution after SEC separation. Given NR's molecular dimensions (∼ 1.5 nm), the observation is consistent with occupancy of a single guest molecule within a cavity or at an inter‐domain interface of the folded protein chain.

**TABLE 1:** Structural parameters from SEC–SAXS for βNaCn, βSCNP and βSCNP‐NR.

Sample	Guinier *R*_g_ [nm], (Std)^a^	P(r) *R*_g_ [nm]^b^	DAMMIF *R*_g_ [nm]^c^	ν^d^	MMF *R*_g_ [nm]^e^	MMF ν^e^
βNaCn	5.3 (±0.5)	4.8	5.0	0.58	5.4	0.60
βSCNP	3.0 (±0.2)	3.0	3.0	0.37	3.1	0.36
βSCNP‐NR	3.8 (±0.1)	3.7	3.5	0.41	3.8	0.43

Radii of gyration (*R*_g_) determined by:

^a^ Guinier analysis (Figure S11)

^b^ real‐space pair distance distribution function P(r)

^c^ ab initio reconstruction (DAMMIF) for the monomeric elution peaks during SEC‐SAXS

^d^ scaling exponent ν calculated from the SEC‐SAXS scattering curves (Figure 2B)

^e^ *R*_g_ and ν from molecular form factor (MMF) analysis of SAXS data, see Figure S19 [39].

**FIGURE 3:**
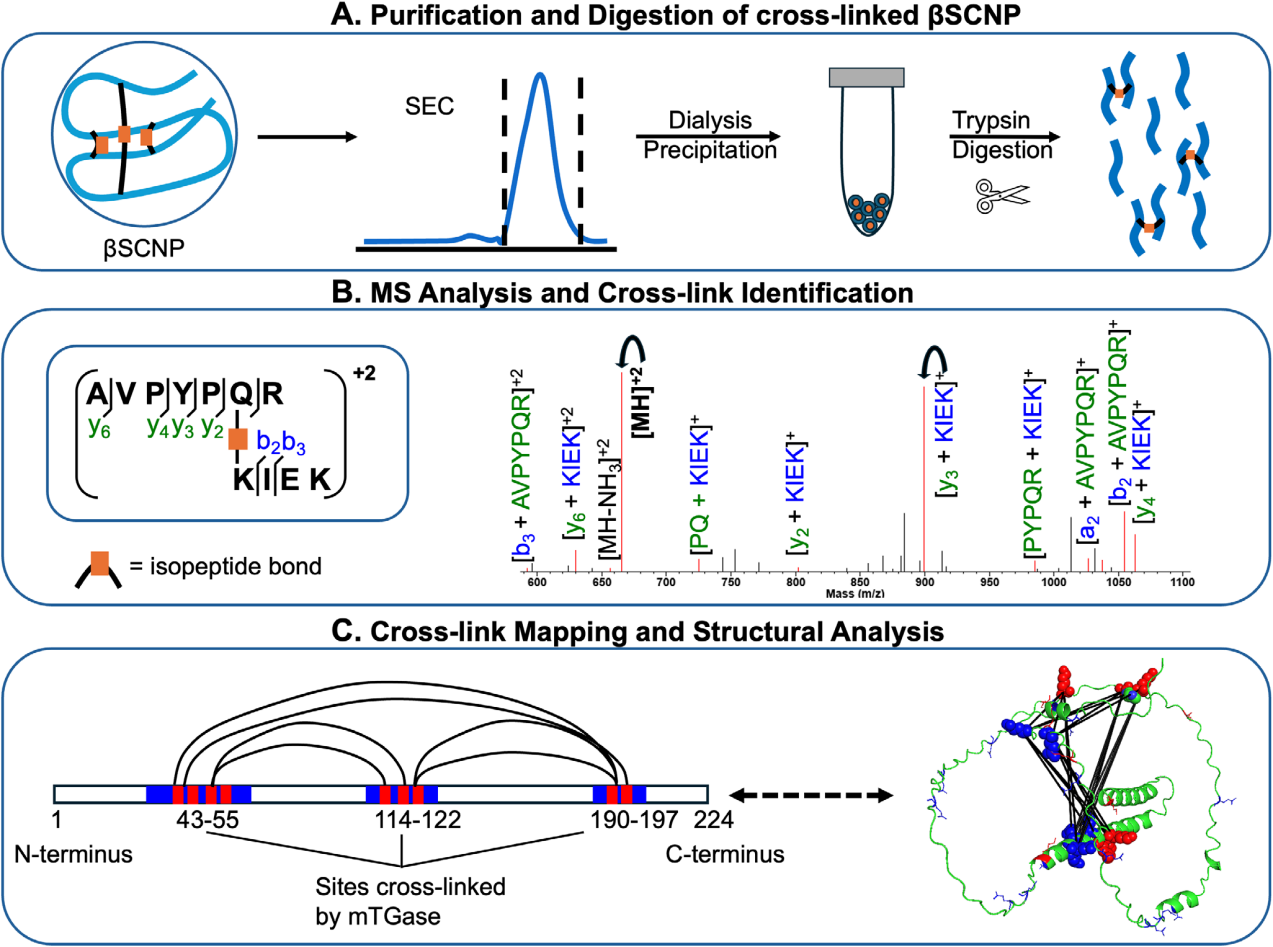
XL‐MS resolves the residue‐level constraint map installed by mTGase onto β‐casein. (A) Preparative SEC separation for the purification of βSCNP to ensure the presence of monomeric species, followed by enzymatic digestion with trypsin to cleave the protein into peptides amenable for MS. (B) Liquid chromatography‐tandem mass spectrometry (LC MS/MS) analysis of digested peptides and database search for cross‐linked peptides against canonical amino acid sequence of β‐casein, UniProt ID P02666. A representative MS/MS spectrum of a valid cross‐linked peptide AVPYPQR‐KIEK (K_44–_Q_197_) with parent ion [MH]^+2^ at *m/z* 665.7935 and its subsequent fragmentation ions annotated in the spectrum. See Figures S14 and S15 and Table S6 for additional spectra. (C) Sequence mapping of XL‐MS identified Gln–Lys isopeptide hotspots; long‐range constraint topology highlights dominant N‐/C‐terminal to mid‐chain linkages, which are superimposed on the high‐resolution three‐dimensional structure of β‐casein and used to define covalent constraints for MD simulations.

To distinguish a collapsed random coil from encoded topology, we next identified the localisation of mTGase‐induced covalent constraints. Cross‐linking mass spectrometry (XL‐MS) reveals that βSCNP formation is dominated by reproducible long‐range Gln–Lys isopeptide hotspots connecting Lys and Gln residues near the N‐terminus (43–55) and C‐terminus (190–197) to those centred around the mid‐chain region (114–122) (Figure 3B,C). XL‐MS identified 9 unique intramolecular Gln–Lys isopeptide cross‐links in βSCNP (detailed cross‐links in Figures S14 and S15, Table S6), with peptide identification false discovery rate (FDR) ≤ 1% and spectral intensity‐based matched spectral intensity > 40% counts per cross‐link. Critically, all 9 identified cross‐links are intramolecular (connecting distinct regions within a single β‐casein chain), thus mTGase‐catalysed topology encoding occurs through intramolecular chain compaction rather than multichain association or oligomerisation (see Supporting Information Section 3(g) for cross‐link validation criteria). One representative mTGase‐cross‐linked dipeptide linking the β‐Cn chain at K_44_–Q_197_ was identified with charge +2 at the full MS (MS1) level, and its fragmentation pattern is annotated in the corresponding MS/MS (MS2) spectrum (Figure 3B) [40, 41, 42]. These are precisely the constraints expected to have topological impact: long‐range intrachain anchors that act as graph‐level edits reshaping the end‐to‐end distance distribution and promoting multiloop collapse pathways, unlike local links that would primarily stiffen short segments [43].

**FIGURE 4:**
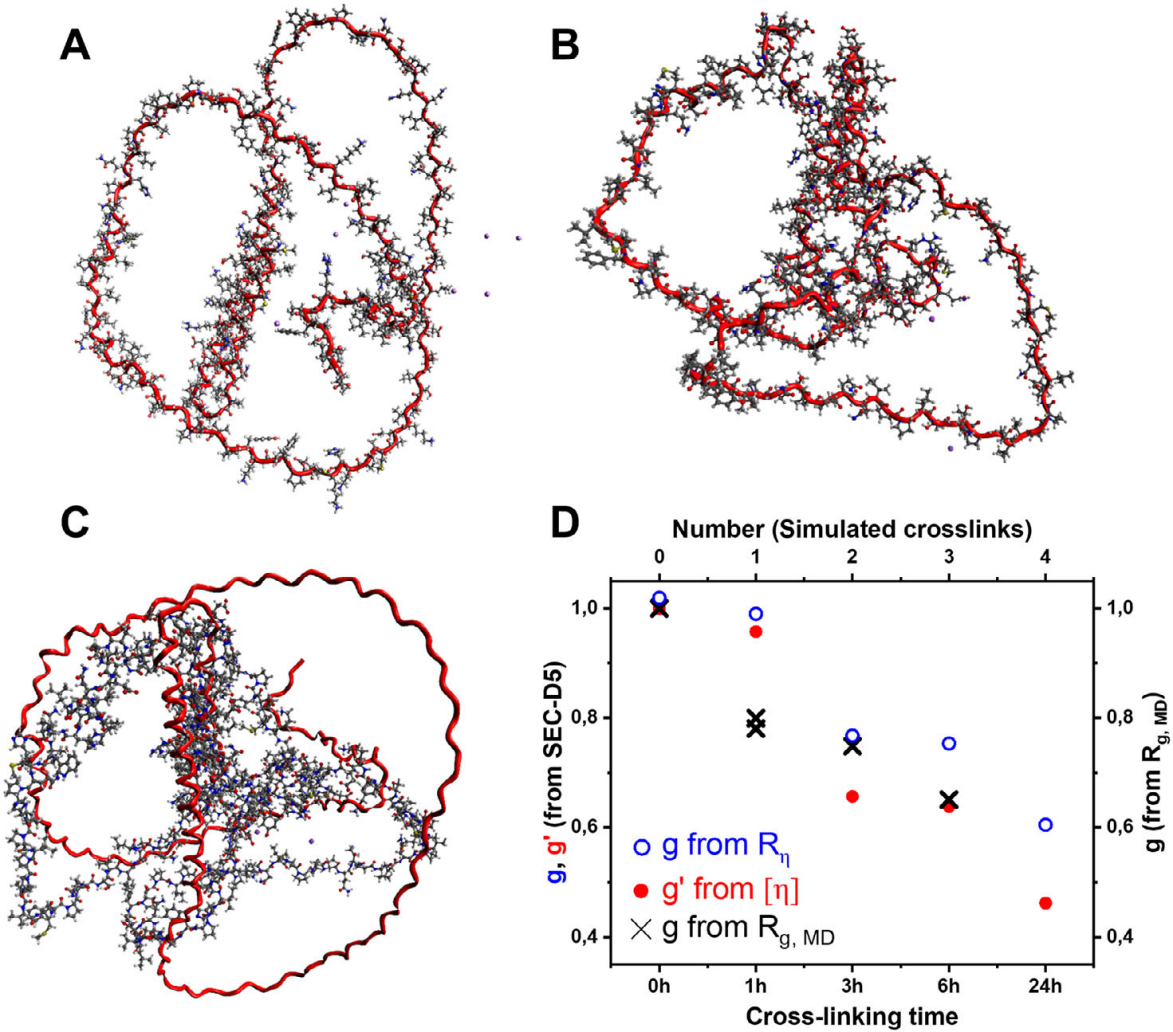
Constraint sufficiency: MD supports an encoded, multi‐loop topology consistent with experiment. (A) MD snapshot of the pristine β‐casein model (P02666). (B) MD snapshot of βSCNP constrained by three XL‐MS‐identified isopeptide links, yielding a loop‐partitioned (“figure‐eight‐like”) topology with partially enclosed pockets. (C) Superposition of the pristine chain (red ribbon) onto the constrained βSCNP skeleton illustrates the topology change induced by sparse long‐range constraints. (D) Experimentally determined contraction factors g (from *R*_η_, open symbols) and g’ (from [η], filled symbols) are compared with compaction inferred from MD‐derived *R*_g_ (crossed symbols), supporting that ∼3 to 4 isopeptide bonds are sufficient to account for the experimentally observed scale of compaction depending on the duration of cross‐linking (see, Equations S12 and S13).

To complement the experimental data, we performed all‐atom MD simulations (Figure 4A–C) in which experimentally identified constraints were implemented individually and in combination (Table S7) [44, 45]. The representative conformations illustrating the structural impact of imposed constraints (Figure S17) demonstrate progressive compaction with each added long‐range anchor, yielding *R*_g_ values in the same order of magnitude as the experimentally observed βSCNP scale; the triple‐constraint model reaches *R*_g_ = 3.0 nm (Table 1). Structurally, the constrained ensembles adopt a multi‐loop (qualitatively “figure‐eight‐like”) topology with partially enclosed pockets (Figure 4B), providing a plausible physical basis for the SAXS response of a protein with locally compact portions of the peptide chain connected by flexible segments [46]. A qualitative comparison of MD‐derived contraction with the SEC‐D5 contraction factors g and g′ (Figure 4D) was used as an orthogonal constraint‐sufficiency analysis, rather than as a kinetic model. The SEC‐D5 time dependence reflects the combined outcome of enzymatic bond formation and ensemble reorganisation. In general, the contraction factors correlate well. The lowest experimental g and g’ values (0.46–0.60) after 24 h incubation likely overestimate the degree of contraction because the precursor was measured under denaturing conditions, which extend its initial dimensions. Within this scope, the MD results support the conclusion that a sparse subset of approximately 3 to 4 long‐range isopeptide bonds is sufficient to account for the experimentally observed compact state after 24 h incubation, further supporting the complementarity of the experimental and computational analyses. Taken together, the XL‐MS constraint map, the MD constraint‐sufficiency analysis, the SEC‐D5 and SEC‐SAXS measurements support the conclusion that the outcome is not a single rigid fold but a topologically biased ensemble. In other words, this is the regime where disordered macromolecules can encode structure without ordered secondary elements.

The ability of βNaCn to undergo enzymatic topology encoding suggests that other intrinsically disordered proteins with comparable Gln/Lys content may be similarly programmable. Supporting SEC‐D5 data (Figures S8–S10; Table S4) for additional casein fractions—commercial NaCn (cNaCn, containing the natural amount of α_s_‐, β‐ and κ‐Cn) and ακ‐Cn‐rich NaCn (ακNaCn, residual from βNaCn purification section S2)—indicate that mTGase‐induced intramolecular contraction is not unique to βNaCn, as analogous contraction was also observed for the corresponding cSCNP and ακSCNP systems. Given the emerging biomedical use of mTGase (e.g., corneal collagen stiffening) [47], these results highlight the broader potential of enzymatic constraint writing, while emphasizing that topology‐resolved characterisation is essential since function cannot be inferred from size alone [46].

## 3 Conclusion

In summary, mTGase‐catalysed isopeptide formation provides a mild route to encode cavity‐containing topology in a disordered single chain while preserving local disorder. Using β‐casein as a stringent IDP‐like substrate, we show that sparse enzymatic long‐range constraints are sufficient to generate a compact single‐chain nanoparticle characterised by strong unimolecular hydrodynamic contraction (g ≈ 0.60, g' ≈ 0.46), residue‐resolved intramolecular cross‐link hotspots and guest‐responsive redistribution of internal density. The key advance is the combination of enzymatic constraint installation, XL‐MS hotspot mapping, and orthogonal topology validation in a genuinely disordered single‐chain protein. More broadly, this workflow provides a route to connect residue‐level covalent editing to ensemble‐level topology in the regime where connectivity, rather than secondary structure, is the primary design lever.

## Conflicts of Interest

The authors declare no conflicts of interest.

## Supporting information



The Supporting Information provides detailed descriptions of sample preparation, analytical instrumentation and experimental methodologies, together with the relevant theoretical background for SEC‐D5 and SAXS. Additional tables and figures from size‐exclusion chromatography, cross‐linking mass spectrometry‐based proteomics and molecular dynamics simulations that support the results in the main manuscript are included.
**Supporting File**: anie72846‐sup‐0001‐SuppMat.pdf.

## Data Availability

The data that support the findings of this study are available from the corresponding author upon reasonable request.
