# Supplementary material for: Enzymatic Encoding of Topology in an Intrinsically Disordered Single‐Chain Protein
Source: Angew Chem Int Ed Engl. 2026 May 26;65(28):e3738676. doi: 10.1002/anie.3738676 (PMC13340486; doi:10.1002/anie.3738676)
Supplement: Supplementary file 1 — The Supporting Information provides detailed descriptions of sample preparation, analytical instrumentation and experimental methodologies, together with the relevant theoretical background for SEC‐D5 and SAXS. Additional tables and figures from size‐exclusion chromatography, cross‐linking mass spectrometry‐based proteomics and molecular dynamics simulations that support the results in the main manuscript are included. Supporting File: anie72846‐sup‐0001‐SuppMat.pdf. [file ANIE-65-e3738676-s001.pdf]

Supplementary Information

Enzymatic Encoding of Topology in an Intrinsically Disordered Single-Chain Protein

Joshua Johani, Kristin Eichelberger, Olga Guskova, Simbulele Charlotte Dunjwa, Hans Bolinsson, Anna-Maria Börjesdotter, Lars Nilsson, Doris Jaros, Harald Rohm, Albena Lederer*

Table of Contents

S1. Chemicals and materials 2
S2. Sample preparation and synthesis 3
(a) Microbial transglutaminase (mTGase) activity assay 3
(b) Purification of β-casein-rich sodium caseinate (βNaCn) 3
(c) Synthesis of single-chain casein nanoparticles (SCNP) 4
(d) Topology read-out by Nile red fluorescence 5
S3. Instrumentation and methods 5
(a) Measurement of the Refractive Index Increment (dn/dc) 5
(b) SDS-PAGE measurements 6
(c) Quintuple detection size exclusion chromatography (SEC-D5). 6
(d) Circular dichroism spectroscopy 6
(e) FTIR Spectroscopy 7
(f) SEC coupled with Small Angle X-ray scattering (SEC-SAXS) 7
(g) Cross-linking mass spectrometry-based bottom-up proteomics (XL-MS) 7
(h) Molecular dynamics simulations 9
(i) *Ab initio* electron density reconstruction from SAXS data with DENSS 10
S4. Supporting theory 10
(a) Basic theory supporting SEC-D5 10
(b) Basic theory supporting SEC-SAXS 12
S5. Supporting results – figures and tables 14

S1. Chemicals and materials

The chemicals used in the study are captured in Table S1 below

Table S1. *The list of chemicals used in the study.*

Chemical	Formula/ Trade name	Source
1,4-dithiothreitol	DTT	Carl Roth GmbH & Co. KG, Karlsruhe, Germany
3-[(3-cholamidopropyl) dimethylammonium]-1-propanesulfonate	CHAPS	Carl Roth GmbH & Co. KG, Karlsruhe, Germany
Anhydrous acetic acid		Merck KGaA, Darmstadt, Germany
Boric acid	H_3BO_4	Carl Roth GmbH & Co. KG, Karlsruhe, Germany
Casein sodium salt	cNaCn	Sigma-Aldrich Chemie GmbH, Steinheim, Germany
Copper sulphate pentahydrate	$CuSO_4.5H_2O$	Carl Roth GmbH & Co. KG, Karlsruhe, Germany
Deionised water		lab Millipore purification system
Hydrochloric acid (37 %)	HCl	Merck KGaA, Darmstadt, Germany
Hydroxylamine hydrochloride	$NH_2OH.HCl$	Sigma-Aldrich Chemie GmbH, Steinheim, Germany
Iron (III) chloride	$FeCl_3$	Carl Roth GmbH & Co. KG, Karlsruhe, Germany
L-glutamic acid γ-monohydroxamate		Sigma-Aldrich Chemie GmbH, Steinheim, Germany
L-glutathione-reduced		Carl Roth GmbH & Co. KG, Karlsruhe, Germany
Microbial transglutaminase Stabizym TGL-100	mTGase	Stabizym GmbH, Roßdorf, Germany
N-carboxybenzyl-L-glutaminyl-glycine	Z-Gln-Gly	Sigma-Aldrich Chemie GmbH, Steinheim, Germany
Potassium chloride	KCl	Carl Roth GmbH & Co. KG, Karlsruhe, Germany
Potassium sulphate	K_2SO_4	Carl Roth GmbH & Co. KG, Karlsruhe, Germany
Sodium azide	NaN_3	Sigma-Aldrich Chemie GmbH, Steinheim, Germany
Sodium chloride	NaCl	Carl Roth GmbH & Co. KG, Karlsruhe, Germany
Sodium hydroxide	NaOH	Sigma-Aldrich Chemie GmbH, Steinheim, Germany
Sodium phosphate dibasic	Na_2HPO_4	Sigma-Aldrich Chemie GmbH, Steinheim, Germany
Sulphuric acid (96 %)	H_2SO_4	VWR International GmbH, Darmstadt, Germany
Tashiro's Indicator		Carl Roth GmbH & Co. KG, Karlsruhe, Germany
Trichloroacetic acid		Carl Roth GmbH & Co. KG, Karlsruhe, Germany
Tris(hydroxymethyl) aminomethane	TRIS	Sigma-Aldrich Chemie GmbH, Steinheim, Germany
Trypsin from bovine pancreas, TCK treated, essentially salt free lyophilised powder, ⩾10000 BAEE U/g protein		Sigma-Aldrich Chemie GmbH, Steinheim, Germany
Urea	$CO(NH_2)_2$	Carl Roth GmbH & Co. KG, Karlsruhe, Germany
α_s-casein	α_s-Cn	Sigma-Aldrich Chemie GmbH, Steinheim, Germany
β-casein	β-Cn	Sigma-Aldrich Chemie GmbH, Steinheim, Germany
κ-casein	κ-Cn	Sigma-Aldrich Chemie GmbH, Steinheim, Germany

S2. Sample preparation and synthesis

(a) Microbial transglutaminase (mTGase) activity assay

mTGase (Stabizym GmbH, Roßdorf, Germany) was dissolved at 5 µL/mL in 0.2 mol/L TRIS-acetate buffer (pH 6.0) and blended 1:2 (v/v) with substrate reagent (0.01 mol/L L-glutathione, 0.1 mol/L hydroxylamine hydrochloride, 0.03 mol/L Z-Gln-Gly in TRIS-acetate buffer; pH 6.0). The samples were incubated in water baths within the 5–50 °C temperature range, followed by blending 1:1 (v/v) with $FeCl_3$ reagent (0.31 mol/L $FeCl_3$ in 0.1 mol/L HCl, 120 mL/L HCl, and 0.73 mol/L trichloroacetic acid in a volumetric ratio of 1:1:1) to stop the enzymatic reaction and to induce a colour reaction of hydroxamate.

$$A_{mTGase}\left[{}^{U}/_{mL}\right] = \frac{c_H \times V_{rxn} \times 1000}{t \times c_E \times V_E} \qquad \text{S1}$$

Where c_H (µmol/mL) is the concentration of hydroxamate formed, V_{rxn} (mL) is the total volume of reaction solution, c_E (mg/mL) and V_E (mL) are the concentration and the volume of enzyme solution respectively.

Hydroxamate concentration was determined by absorbance at λ = 525 nm (Ultrospec 8000, GE Healthcare Europe GmbH, Freising, Germany) against a relative calibration line of L-glutamic acid γ-monohydroxamate in a concentration range of 0.0005–0.0025 mol/L. A control experiment using the reagents but excluding mTGase was used as a blank. The unit for enzyme activity is Units (U) per mL, where 1 U corresponds to 1 µmol hydroxamate formed during 1 min of enzymatic reaction, according to the equation above. The results reported are mean values from triplicate experiments.

(b) Purification of β-casein-rich sodium caseinate (βNaCn)

Low-heat skim milk powder (Sachsenmilch Leppersdorf GmbH, Leppersdorf, Germany) was reconstituted in demineralised water at a concentration of 100 g/kg dry matter, with 0.3 g/kg sodium azide (NaN_3) added as a bacteriostatic agent. The reconstituted skim milk (RSM) was held in a water bath at 1 °C for at least 48 hours to promote the dissociation of β-casein from the casein micelles. Separation was performed using cross-flow microfiltration with a 0.1 µm polyethersulfone membrane (Sartorius AG, Göttingen, Germany). To improve separation efficiency, the retentate volume was replenished with chilled demineralized water, and the process was repeated ten times per day for 3 days while maintaining the retentate at 1 °C. After warming to room temperature, both the permeate and retentate were acidified to pH 4.6 using 6 mol/L HCl, causing the casein to precipitate. The precipitated casein was separated from the whey by filtration through cellulose filters (Rotilabo type 600P, Carl Roth GmbH + Co. KG, Karlsruhe, Germany), washed extensively with demineralized water, removed from the filters, and freeze-dried (Alpha 1-4 LD Plus, Martin Christ GmbH, Osterode am Harz, Germany). The resulting permeate and retentate powders contained crude protein contents of 952 g/kg and 949 g/kg, respectively, and are referred to as β-casein-rich sodium caseinate (βNaCn) and a residual ακ-casein-enriched sodium caseinate fraction (ακNaCn). The process is summarised in Scheme S1 below.

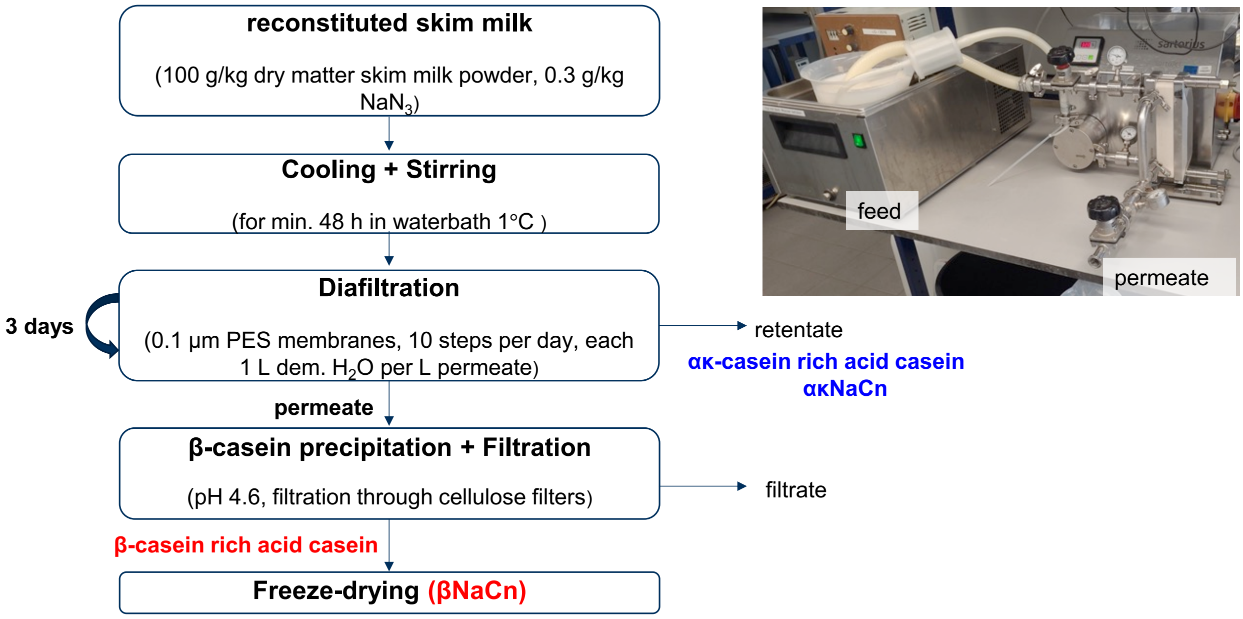

Scheme S1. Set-up of the cross-flow microfiltration process for the preparation of βNaCn and ακNaCn.

(c) Synthesis of single-chain casein nanoparticles (SCNP)

Caseinate solutions for SCNP preparation were made by dissolving c-, β- and ακNaCn at a concentration of 0.5 g/kg at neutral pH. The samples were temperature equilibrated at 40 °C before adding the required amount of mTGase 3 U/g protein estimated from the enzyme activity (Figure S2). Incubation was carried out in a water bath at 40 °C and the enzyme was inactivated after predefined periods of time (0 – 24 h) by heat treatment in a water bath at 85 °C for 15 min and subsequent cooling in ice water (Scheme S2). A reference sample without enzyme addition was treated in the same way. The samples were concentrated by rotational vacuum evaporation (Heidolph Instruments GmbH & Co. KG, Schwabach, Germany) at 70 °C. Upon cooling in ice-water the samples were then acidified with 1 mol/L HCl to pH 4.6 to precipitate the c-, β- and ακSCNPs. After centrifugation at 3000 g for 3 min, the precipitate was washed with deionized water, suspended in a minimum amount of water followed by neutralisation to pH ~7 with 1 mol/L NaOH and finally freeze-dried (Martin Christ Gefriertrocknungsanlagen GmbH, Osterode am Harz, Germany).

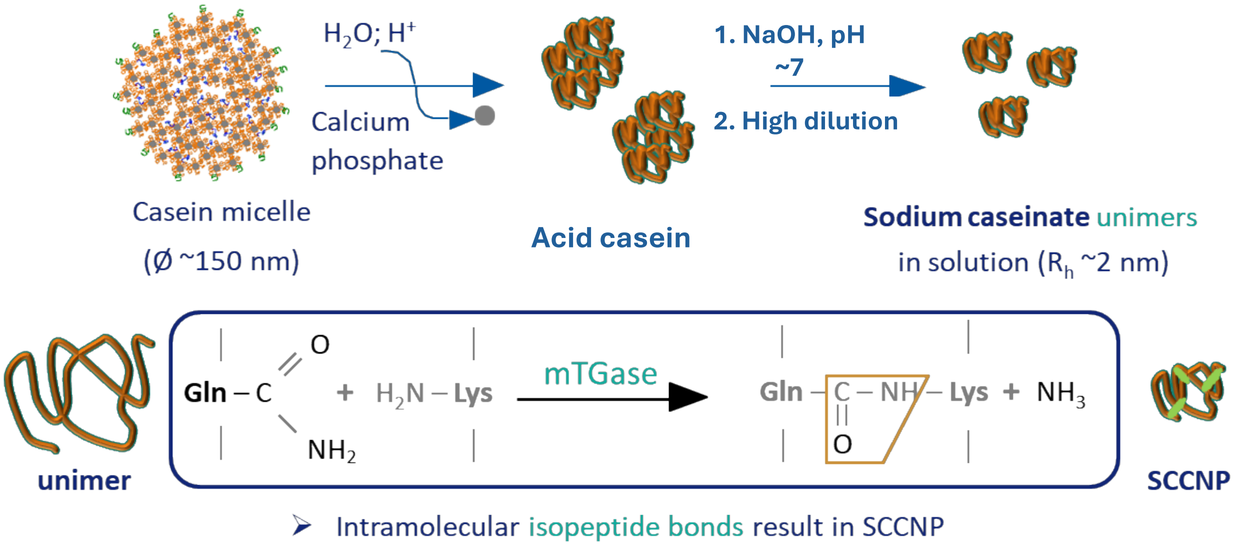

Scheme S2. Synthesis of single-chain casein nanoparticles through enzyme catalysed intramolecular cross-linking with mTGase.

(d) Topology read-out by Nile red fluorescence

For the encapsulation of hydrophobic dyes into the SCNPs, 100 µL of dye solution (1 mg/mL Nile Red (NR)) in methanol was transferred to glass vials and methanol was evaporated to obtain thin films. Then 20 mL of cNaCn, βNaCn, cSCNP, βSCNP solutions (0.5 mg/mL) were added to the vials and stirred overnight under exclusion of light. The samples after dispersion of NR were cNaCn+NR, βNaCn+NR, cSCNP+NR, and βSCNP+NR. Another sample set of cNaCn and βNaCn was incubated at 40 °C for 24 h with 3 U/g mTGase to make cNaCn-NR and βNaCn-NR. After removal of any free dye by centrifugation (3000 rpm for 2 min), testing for the loaded dye was carried out by fluorescence spectroscopy and UV-vis absorbance analyses of the cSCNP and βSCNP solutions.

S3. Instrumentation and methods

(a) Measurement of the Refractive Index Increment (dn/dc)

Batch measurement.

The determination of the specific dn/dc of casein was performed in batch mode in the same solvent as employed for the SEC-D5 experiments (6 mol/L urea at pH 6.8, containing 0.1 mol/L NaCl, 0.1 mol/L Na_2HPO_4 and 0.002 mol/L CHAPS) at 25 °C using the Optilab® T-rEX refractive index detector off-line. This technique was applied to cNaCn because it contains all the four components of casein — α_{s1}-, α_{s2}-β- and κ-Cn, allowing for more accurate batch dn/dc determination of the average protein material. Five different concentrations in the range where a linear relationship of refractive index response vs concentration is true are usually required. We manually injected five casein concentrations from 0.2-3 mg/mL in the order from the lowest to the highest for the dn/dc measurements, but we disregarded the 0.2 mg/mL measurements because of poor reproducibility and low signal-noise ratio. The reported results are therefore based on a four-point batch determination of the dn/dc in duplicate, evaluated with the procedure dn/dc from RI (software Astra 8.1.2, Wyatt Technology, USA).

Online measurement.

Online determination of the dn/dc of casein in urea buffer was carried out by integration of the dRI peak area of βNaCn in the SEC chromatograms, allowing for more accurate dn/dc determination of casein

samples laden with possible contamination of salts from the preparation process. A high mass recovery was observed for the βNaCn. The Astra 8.1.2 (Wyatt Technology, USA) software method dn/dc from peak assumed 100 % sample mass recovery to evaluate the data received from the hyphenated Optilab® Neon (Wyatt Technology, US) refractive index detector. An average from three measurements was determined for the dn/dc values.

(b) SDS-PAGE measurements

Sodium dodecyl sulfate polyacrylamide gel electrophoresis (SDS-PAGE) was performed using a vertical XCell SureLock™ Mini-Cell system from (Invitrogen, Carlsbad, CA, USA), with Novex™ WedgeWell™ 12 % Tris-Glycine gels (Thermo Fisher Scientific, Waltham, MA, USA). 40 µL of the cross-linked and inactivated sodium caseinate solutions with protein concentration of 0.5 g/kg were mixed with 20 µL deionised water and 20 µL sample buffer (2x Laemmli buffer concentrate, Sigma-Aldrich, St. Louis, MO, USA) and heated for 5 min at 100 °C in a water bath. A solution of 0,5 g/kg pure β-casein (purity: ≥ 98 %; Sigma Aldrich Chemie GmbH, Steinheim, Germany) was used as a standard and treated in same way. Ten µL of the mixture or protein size standard (PageRuler Unstained Protein Ladder #26614, Thermo Fisher Scientific, Henningsdorf, Germany) were loaded onto the gel. SDS-PAGE was carried out at 120 V using an 1:20 MOPS buffer (209.2 g/L MOPS, 121.2 g/L Tris, 20.0 g/L SDS, 6.0 g/L EDTA dissolved in deionised water; all from Carl Roth GmbH + Co.KG, Karlsruhe, Germany). Protein fractions were visualised by staining with Coomassie Brilliant Blue G250 (0.6 mg/L in deionised water with 3 mL HCl; Merck KGaA, Darmstadt, Germany) for 30 min, destained in deionised water for approximately 3 h with several water changes.

(c) Quintuple detection size exclusion chromatography (SEC-D5).

Samples for chemical analyses were dissolved in the urea buffer for protein unfolding and enzyme inactivation and were measured within one day. The samples were filtered through 0.2 µm RC filters and 100 µL were injected for analysis. The SEC system consisted of an Agilent Infinity II HPLC with an isocratic pump, inline degasser, thermostated autosampler and multiwavelength detector. The pump delivered Urea buffer pH 6.8 (6 M urea, 100 mM Na_2HPO_4, and 100 mM NaCl filtered through Millipore 0.2 µm CA membrane) at an isocratic flowrate of 0.5 mL/min through the Cytiva Superose Increase 10/300 GL + Cytiva Superdex Increase 10/300 GL SEC columns in series to the Wyatt Neon detectors Dawn MALS WD3-01 with inbuilt QELS (λ 663.8 nm), Viscostar 291-V2 and Optilab dRI WOP1-02 (λ 658 nm). The HPLC was controlled by Wyatt Technologies Vision Run v3.1.1.9 and the data were collected and processed using Wyatt Technologies Astra v 8.1.2 software. Molar mass was calculated with a 1st order Berry fit using either dRI (dn/dc = 0.140 mL/g) or UV absorbance at 280 nm (ε = 0.734 mL/mg).

(d) Circular dichroism spectroscopy

CD spectra were recorded on an Applied Photophysics Chirascan Plus CD spectrometer in the range of 190–250 nm in 0.15 mL quartz cuvettes with the optical path length of 0.01 cm at a protein concentration of 0.5 mg/mL. Spectra of the solvent were subtracted from the protein spectra. The spectra were recorded at ambient temperature after equilibrating the sample for 20 min. Absorption by mTGase in solution was negligible because of its very low concentration.

(e) FTIR Spectroscopy

FTIR analyses were performed in the wave number range of 4000–600 cm^{-1} with spectral resolution 4 cm^{-1}, using a Bruker Vertex 80v spectrometer (Bruker Optics GmbH, Ettlingen, Germany) equipped with mercury-cadmium-telluride detector (InfraRed Associates Inc., Stuart (FL), USA) and attenuated total single reflection (ATR) Golden Gate diamond unit (Specac, Orpington, UK). 100 scans were accumulated for every spectrum. A baseline correction was applied for every spectrum using standard tools of spectroscopic software OPUS 7.5 (Bruker Optics GmbH, Ettlingen, Germany) as the post-processing prior to spectra's comparison. Both pristine sample of βNaCn and βSCNP were measured.

(f) SEC coupled with Small Angle X-ray scattering (SEC-SAXS)

The fractionation of βNaCn, βSCNP and βSCNP-NR solutions was performed on an 1100-series HPLC from Agilent Technologies, Santa Clara, USA. 6 mol/L urea buffer at pH 6.8 containing 0.1 mol/L NaCl and 0.1 mol/L Na_2HPO_4 was the carrier liquid delivered through an in-line degasser at a flow rate of 0.5 mL/min to a Cytiva Superdex 200 Increase 10/300 GL SEC column by an isocratic pump. An autosampler was used to inject the samples, and the SEC column was connected to a UV detector set at 280 nm, whose outlet was connected directly to the sample flow-through quartz capillary of the synchrotron source via an on-line multiangle light scattering detector (MALS, Heleos II, Wyatt Technology). ASTRA software (Wyatt Technology) was used for the acquisition of UV and MALS data. SAXS measurements were carried CoSAXS beamline at the MAX IV laboratory, Lund, Sweden. The high X-ray flux, 1013 photons/s at 12.4 keV, and a nominal wavelength of λ = 0.99 Å. The data was collected on an Eiger2 4 M (Dectris) detector within an evacuated flight tube, positioned 3.5 m from the sample position. The detector at this position covers a q-range of $0.003 < q < 0.30$ $Å^{-1}$, where q is expressed by equation S14. The flow cell consists of a 0.98 mm inner-diameter quartz capillary, with a 10 µm wall thickness. The acquisition rate during SEC-UV-SAXS fractionation was set to 1 frame/s.

The ATSAS program suite was used for the evaluation of SEC-SAXS data. The frames of experiments obtained from SEC-SAXS were displayed in the CHROMIXS program, in which interactive tools for locating buffer and sample regions were used. The Guinier analyses of R_g, pair-distribution functions (P(r)), Porod volume and Kratky analyses were enabled by the PRIMUS package. Reduced SAXS data was transferred to SASview and fitted to form factor models of a coil for βNaCn coil, flexible cylinder for βSCNP and ellipsoid revolving about the short axis for βSCNP-NR.

(g) Cross-linking mass spectrometry-based bottom-up proteomics (XL-MS)

Enzymatic hydrolysis.

Lyophilised samples were dissolved in TRIS buffer (0.1 mol/L tris(hydroxymethyl)aminomethane, pH 7.8) to achieve a protein concentration of 1 mg/mL. To this, aliquots of the sample solution were mixed with 0.02 mmol/L trypsin, which had been prepared in 1 mM hydrochloric acid according to the supplier's instructions (Sigma-Aldrich, Steinheim, Germany), maintaining an enzyme-to-substrate ratio of 1:100. The enzymatic hydrolysis was performed at 37 °C for 16 hours and then halted by freezing the samples, followed by lyophilization.

Peptide sequencing via reversed phase high performance liquid chromatography coupled with high resolution tandem mass spectrometry (RP-HPLC-HRMS).

Mass spectrometry was performed using a Thermo Scientific Fusion mass spectrometer equipped with a Nanospray Flex ionization source. The sample was introduced through a stainless-steel nano-bore

emitter. Data were collected in positive mode with spray voltage set to 1.8kV and ion transfer capillary set to 275 °C. Spectra were internally calibrated using polysiloxane ions at m/z = 445.12003. MS1 scans were performed using the orbitrap detector set at a resolution of 240 000 over the scan range 375-1500 with Automatic Gain Control (AGC) target set to standard. MS2 acquisitions were performed using monoisotopic precursor selection for ion with charges +2-+7 with error tolerance set to ±10 ppm. Precursor ions were excluded from fragmentation once for a period of 60 s. Precursor ions were selected for fragmentation in high-energy collision induced dissociation (HCD) mode using the quadrupole mass analyser with HCD energy set to 30 %. Fragment ions were detected in the Orbitrap mass analyser set to a resolution of 50 000. The AGC target was set to standard and the maximum injection time to 100 ms. The data was acquired in centroid mode and was reported in *.raw format.

Search for lysine-glutamine (K-Q) isopeptide cross-links

UCSF Protein Prospector bioinformatics software.

The *.raw data files generated by the Orbitrap Fusion Lumos Tribrid were converted into *.mgf format using MSConvert (Version 3.0.23233-c72ce16, ProteoWizard Tools). These *.mgf files were analysed through the Batch-Tag Web tool on the Protein Prospector website. The analysis involved a combination of mass spectrometry data, database searches, and manual evaluation to confirm or reject candidate cross-linked peptide pairs in a complex protein mixture. Experimental adjustments were made based on methods from existing literature, tailored to casein-specific analysis.

The Batch-Tag Web form was configured with the following parameters:

- **Database**: UniProtKB (version 2020.09.02).
- Taxonomy Filter: Bos taurus.
- **Pre-search Filters**: Accession numbers P02662, P02663, P02666, and P02668.
- Precursor Charge Range: 2 to 5.
- Parent Ion Tolerance: 5 ppm.
- Fragment Ion Tolerance: 10 ppm.
- **Digestion Enzyme**: Trypsin, allowing up to 3 missed cleavages.
- Constant Modifications: None.
- **Variable Modifications**: Oxidation of methionine (Met) and phosphorylation of serine (Ser), threonine (Thr), and tyrosine (Tyr).
- Mass Modifications Range: -18 to 4000 Da.
- **Cross-Link Definition**: Amino acid link between lysine (K) or protein N-terminus to glutamine (Q), with a bridge element composition of N-1 H-3.
- **Instrument Settings**: High-resolution ESI-Q-Orbitrap with HCD MS/MS.

The analysis relied on the percentage of matched intensity (% matched intensity) provided by Protein Prospector's Batch-Tag/Search Compare tool. For cross-linked peptides to be accepted, they needed to meet two criteria:

1. **% Matched Intensity**: Greater than 40%.

2. **Score Differential**: Positive value, indicating that the spectra matched the cross-linked dipeptide more closely than any other linear peptide from the protein.

These criteria ensured reliable identification of cross-linked peptides within the experimental dataset.

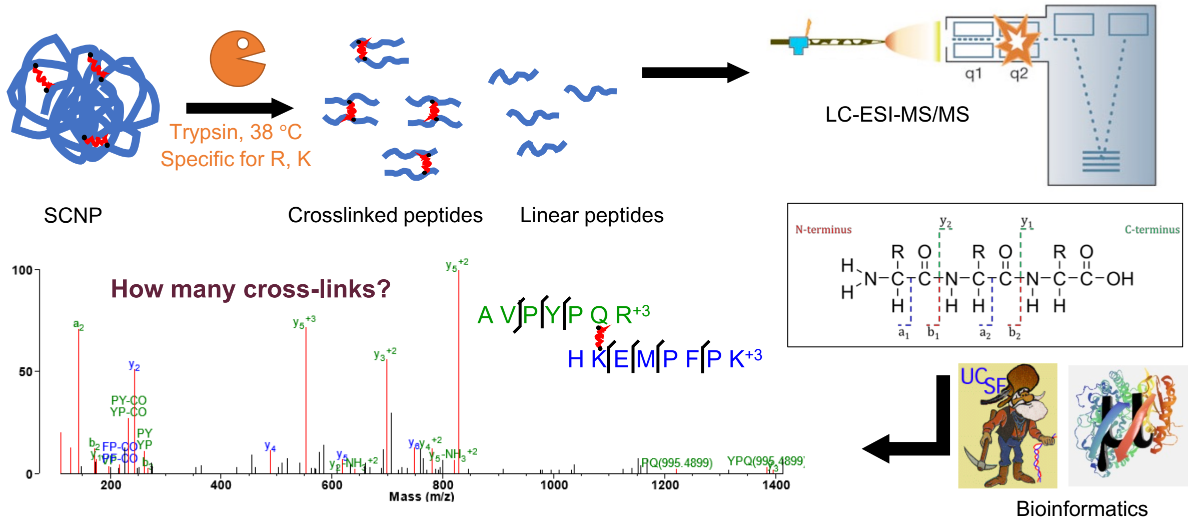

Scheme S3. Summary of workflow for the search and identification of isopeptide cross-links in βSCNPs via bottom-up cross-linking mass spectrometry proteomics.

MeroX/StavroX bioinformatics software.

*.raw MS/MS data files converted to *.mgf in the previous section and *.fasta files containing the casein protein sequences were provided to the software MeroX/StavroX. The software loaded these files through the "Load Fasta" and "Load MS-File" options, decoding key data such as m/z-value, charge state (z), scan number, retention time, and the MS/MS spectrum. Trypsin was entered as the protease, and the Lys box was checked for blocked as a cross-linking site to exclude terminal Lys as cross-linked candidate. We created a profile for mTGase as the cross-linker by leaving the composition field blank. The enzyme effects a mass difference of 0, the isopeptide cross-link between K-Q residues was included as '-NH3'. and technical details of the mass spectrometric analysis. Using the provided protein sequences and experimental settings, the software predicted all possible peptide combinations and matched these to the precursor masses in the MS file. The software then validated the identified candidates by comparing the theoretical fragmentation patterns of the cross-linked peptides with the observed MS/MS spectra.

(h) Molecular dynamics simulations

The predicted structure of β-casein (224 amino acids, UniProt ID: P02666) was downloaded from the AlphaFold Protein Structure Database [https://alphafold.ebi.ac.uk/entry/P02666]. At physiological pH (7.4), β-casein carries a net charge of -7*e*, so seven sodium ions were added to each simulated system to maintain charge neutrality. All simulations were conducted using BIOVIA Materials Studio 9.0 (BIOVIA, Dassault Systèmes, Materials Studio 9.0; San Diego, CA, USA, 2014), employing the polymer consistent force field (PCFF) force field.[9] To model the aqueous environment, an implicit water model with a dielectric constant of 78.3 was used, allowing efficient exploration of conformational space without explicit solvent dynamics. Conformations of each cross-linked structure in salt-free solution were optimised using the Smart algorithm with ultra-fine quality, applying convergence criteria of 2×10^{-5} kcal/mol for energy, 0.001 kcal/mol/Å for force, and 10^{-5} Å for displacement. Partial atomic charges

were assigned per the PCFF force field. Electrostatic interactions were treated using the Ewald summation method with a cut-off distance of 12.5 Å (spline width 1 Å, buffer width 0.5 Å), employing cubic spline truncation to accurately simulate Coulombic interactions, especially of the counter-ions added for system neutrality. For van der Waals interactions, the atom-based summation method was applied. Equilibration was performed via a 30 ps and productive MD run via a 3ns MD run in the NVT ensemble, ensuring a stable temperature of 300 K. A sufficiently large cubic simulation box was used to prevent periodic self-interactions of the protein. The simulations are not intended to sample the full conformational ensemble but to probe the structural consequences of specific constraint sets.

Intrachain distance scaling was analysed using the atomistic structures obtained from MD simulations. For each system, Cα coordinates of all residues (i = 1…N) were extracted, and pairwise Euclidean distances were computed: $|Ri - Rj| = ||Ri - Rj||$, where Ri and Rj denote the positions of the Cα atoms of residues i and j, respectively. To characterise scaling behaviour, distances were grouped according to sequence separation: $s = |i - j|$. For each value of s, the mean intrachain distance was computed as: $\langle|Ri - Rj|\rangle(s) = (1/Ns)\, \Sigma_{|i-j|=s} |Ri - Rj|$, where Ns is the number of residue pairs with separation s.
The resulting function $\langle|Ri - Rj|\rangle(s)$ was analysed both in linear and double-logarithmic representations (Figure S16). The log-log representation enables identification of an approximate power-law regime: $\langle|Ri - Rj|\rangle \sim s^{v\_app}$, where v_{app} is an apparent Flory exponent.
The v_{app} exponent was obtained by linear regression of $\log\langle|Ri - Rj|\rangle$ versus log(s) over an intermediate range of sequence separations ($5 \leq s \leq 50$). This range was selected to avoid:(1) local chain stiffness effects at small s, and (2) finite-size and long-range correlation effects at large s, which lead to systematic deviations from scaling behaviour (see Figure S16).The resulting v_{app} values quantify relative changes in chain compactness across different cross-linking states. To estimate the uncertainty of the extracted scaling exponents, a bootstrap resampling procedure was applied. The uncertainty in v_{app} was estimated from the standard error of the linear regression in the log-log representation. This reflects the variability of the scaling behavior within the selected intermediate regime.

(i) *Ab initio* electron density reconstruction from SAXS data with DENSS

The GNOM package was used to carry-out Guinier and pair distribution function p(r) analyses. The maximum linear dimension of the molecule, D_{max}, is calibrated for goodness-of-fit by enforcing a smooth zeroing of $P(D_{max})$ using the maximum range of q available in the experiment. DENSS was used to calculate the *ab initio* electron density map directly from the GNOM output. Twenty reconstructions of electron density mapping were performed in slow mode using default parameters and subsequently averaged. The mean real space correlation (RSC) was > 0.95 and the RSC standard deviation was low. The software performed a Fourier Shell Correlation on the averaged electron density map from which the resolution in Å of the reconstructions was estimated. Alignment of the reconstructions to the structure was achieved using the DENSS alignment function in BioXTAS RAW. The reconstructions were visualised using PyMOL Molecular Graphics System, Version 3.1.6.1 with a coloured density ramp called that gradually changes colour and opacity from transparent blue at the lowest densities through cyan, green, yellow and finally red at the highest densities. The colour density ramp can be entered in PyMOL with the script "> *volume_ramp_new colored_density, 0.08 blue 0 0.10 blue 0.025 0.48 green 0 0.50 green 0.025 0.98 red 0 0.99 red 0.025 50 red 0.05*" The sigma (σ) level denotes the standard deviation above the average electron density value of the reconstructed model. A five-sigma level (5σ) contour will envelope a region that has electron density values greater than 5σ over the average value.

S4. Supporting theory

(a) Basic theory supporting SEC-D5

SEC is based on an entropic separation mechanism in which the separation is governed by hydrodynamic volume differences among macromolecules in solution. These size differences are a function of molar mass, polymer chain architecture or topology, and polymer conformation (shape). The largest polymers experience exclusion from the columns pore networks and therefore elute earlier, while the smaller macromolecules experience a longer flow path through the porous network and elute last.

Online SEC coupling with quintuple detection (including UV, MALS, DLS, viscometer and dRI) affords, in a single run, information described by the brief theory that follows subsequently.

MALS measures the intensity (I) of scattered light proportional to the properties of the sample solution according to the Zimm equation S2, where M is the molar mass, c is the concentration of the sample, P(θ) is the particle scattering function, and dn/dc is the specific refractive index increment of the sample in the particular solvent.

$$\mathrm{I}(\theta)_{\mathrm{scattered}} \propto \mathrm{M} \cdot \mathrm{c} \cdot \left(\frac{\mathrm{dn}}{\mathrm{dc}}\right)^2 \cdot \mathrm{P}(\theta) \qquad S2$$

For a polymer with concentration c_i, and molar mass M_i, the number- M_n, weight- M_w, and z-average- M_z molar masses are calculated using equation S3. The polydispersity (*Đ*) is then determined as the ratio in equation S4.

$$M_\theta = \frac{\sum_i c_i M_i^{\,x}}{\sum_i c_i M_i^{\,x-1}} \quad x = 0, \theta = n; x = 1, \theta = w; x = 2, \theta = z \qquad S3$$

$$Đ = \frac{M_w}{M_n} \qquad S4$$

Radius of gyration (R_g) can be calculated from the angular dependence of the intensity of scattered light as described by the particle scattering function P(θ) in equation S5, where q is the scattering vector, λ is the wavelength of the incident light.

$$\lim_{\theta \to 0} P(\theta) = 1 - \frac{16 \cdot \pi^2}{3 \cdot \lambda^2} \cdot R_g^2 \cdot sin^2\left(\frac{\theta}{2}\right) = 1 - \frac{q^2}{3} \cdot R_g^2 \qquad S5$$

R_g determination by MALS is limited to particles with sizes approximately > λ/20 nm because of isotropic scattering at smaller sizes.

In contrast to the radius of gyration, the hydrodynamic radius is calculated form the diffusion coefficient, which is not discussed in this study, but is mentioned here for the sake of completeness. Due to Brownian motion, diffusion coefficient (D) can be measured from the time-dependent fluctuations in the net scattering intensity. D is related to hydrodynamic radius (R_h) of the particle via the Stoke-Einstein equation S6 where k_B denotes the Boltzmann constant, T is the absolute temperature in K and η_0 is the dynamic viscosity of the solvent.

$$\mathrm{D} = \frac{\mathrm{k_B T}}{6\pi\eta_o \mathrm{R_h}} \qquad S6$$

The differential viscometer measures the specific viscosity (η_{sp}) of the solution. Through online coupling the polymer concentration is measured simultaneously so that the intrinsic viscosity [η] which describes how a polymer changes viscosity of the solution can be computed in equation S7:

$$[\eta] = \lim_{c \to 0} \frac{\eta_{sp}}{c} \qquad S7$$

The Einstein-Simha relationship which relates [η] of an analyte to the volume of a hypothetical sphere having the same increment of viscosity as the analyte, thus viscosity radius (R_η) can be calculated through equation S8.

$$R_\eta = \left(\frac{3\,[\eta] M}{10\,\pi\,N_A}\right)^{\frac{1}{3}} \qquad S8$$

The dependencies R_g and [η] on molar mass defined by equations S9 and S10 can be presented in double logarithmic plots. The slopes of the plots, the universal scaling exponent ν based on R_g, and the Kuhn-Mark-Houwink-Sakurada (KHMS) parameter α based on [η], bear information pertaining to polymer conformation summarised in Table S2.

$$R_g = KM^{\nu} \quad S9$$

$$[\eta] = K_{\alpha}M^{\alpha} \quad S10$$

Table S2. Theoretical values defined for polymer conformations which are expected from the scaling exponent ν and the KMHS parameter α.

ν	α	Conformation
1	2	anisotropic rod-like
0.5-0.6	0.5-0.8	linear random coil
0.44-0.5	0.33-0.5	branched polymer, compact
0.33	0	dense sphere

The degree of compactness of a polymer is directly correlated to its mechanical properties such as tensile and compressive strength. Apparent density (d_{app}) is a complementary measure of polymer compactness which can be calculated using eq. S11. R can be either R_g, R_h or R_η, M is the molar mass, N_A is the Avogadro number and in case of R_g a geometrical correction factor $\left(\frac{3}{5}\right)^{\frac{3}{2}}$ is applied.

$$d_{app} = \frac{3M}{4\pi N_A R^3} \quad S11$$

The contraction factor specifically accounts for the reduction in size without any change in molar mass. Initially established for branched polymers, Engelke *et al.* extended its application to single-chain nanoparticles. The radius model contraction factor g is based on R_g (S12) while the viscosity model g' is based on [η] (S13). The values of g and g' are always < 1, with smaller values representing increased contraction.

$$g = \left.\frac{R_{g\,SCNP}^2}{R_{g\,linear}^2}\right|M \quad S12$$

$$g' = \left.\frac{[\eta]_{SCNP}}{[\eta]_{linear}}\right|M \quad S13$$

(b) Basic theory supporting SEC-SAXS

SAXS provides low-resolution structural information on macromolecules and nanoscale particles in solution by measuring the elastic scattering intensity I(q) as a function of the magnitude of the scattering vector – equation S16, where λ is the X-ray wavelength and 2θ is the scattering angle. Under dilute, non-interacting conditions, the scattering is directly proportional to the orientationally averaged particle form factor.

$$q = \frac{4\pi}{\lambda}\sin(\theta), \quad S14$$

Scattering Intensity and Form Factor. For monodisperse particles, the SAXS intensity may be expressed as S14 where ϕ is the particle volume fraction, $\Delta\rho$ the electron density contrast, V the particle volume, and P(q) the normalised form factor. SEC–SAXS minimizes polydispersity and interparticle interactions, allowing S15 to hold across the elution peak.

$$I(q) = \phi(\Delta\rho)^2V^2P(q) \quad S15$$

Low-q Regime: Guinier Approximation. At sufficiently low angles ($qR_g \leq 1.3$), the Guinier approximation describes the scattering as S16, from which the radius of gyration R_g and forward

intensity I(0) can be obtained. Because I(0) ∝ M_w, the Guinier region enables molecular weight estimation in systems where the concentration is known.

$$I(q) = I(0)e^{(-\frac{q^2R_g^2}{3})} \quad \text{S16}$$

Real-Space Representation: Pair-Distance Distribution Function. The overall size and shape of the particle are represented by the pair-distance distribution function p(r), which relates to the scattering intensity through S17 allowing extraction of the maximum particle dimension D_{max} and qualitative shape information.

$$I(q) = 4\pi \int_0^{D_{\max}} p(r) \frac{\sin{(qr)}}{qr}\, dr \quad \text{S17}$$

High-q Regime: Porod Behaviour. For compact particles with well-defined interfaces, the high-q region follows Porod's law S18, and the Porod invariant S19 provides information on total scattering contrast.

$$I(q) \propto q^{-4} \quad \text{S18}$$

$$Q = \int_0^{\infty} q^2 I(q)\, dq \quad \text{S19}$$

Kratky Analysis. The Kratky plot provides a rapid qualitative assessment of particle compactness: well-folded or globular systems show a characteristic bell-shaped maximum, whereas flexible or partially disordered systems exhibit a continuous rise. The Kratky transformation is S20 and the dimensionless Kratky representation, useful for comparing samples of different sizes or conditions, is given by S21.

$$K(q) = q^2 I(q) \quad \text{S20}$$

$$\frac{qR_g}{\sqrt{3}} \text{ vs. } \frac{qR_g\, I(q)}{I(0)} \quad \text{S21}$$

In addition to Eq. S9, the scaling exponent v can also be derived from the Porod region of the double logarithmic SAXS scattering curve as follows:

$$\nu = \frac{-1}{d_{fractal}} = \frac{-1}{slope_{scattering\ curve}} \quad \text{S22}$$

S5. Supporting results – figures and tables

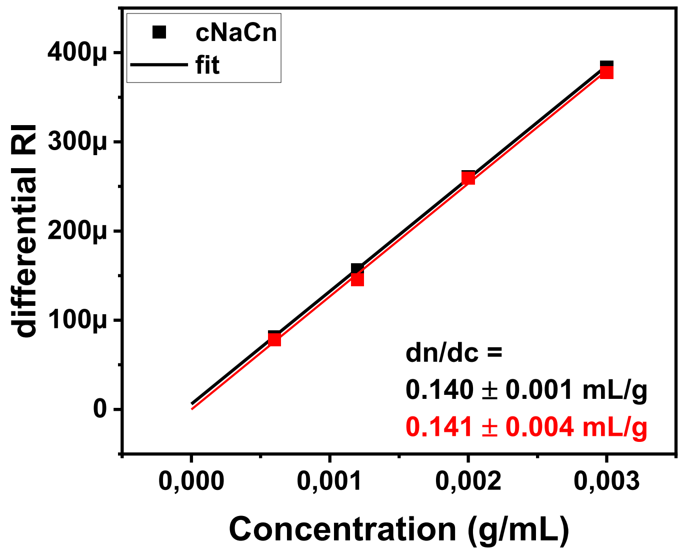

Figure S1: Refractive Index Increment (dn/dc) of cNaCn in 6 M urea buffer at 25 °C, laser wavelength (λ) 658 nm.

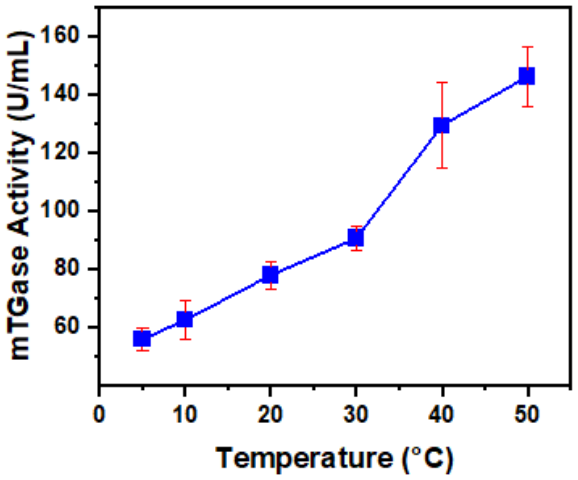

Figure S2. Activity of mTGase at different temperatures in TRIS acetate buffer.

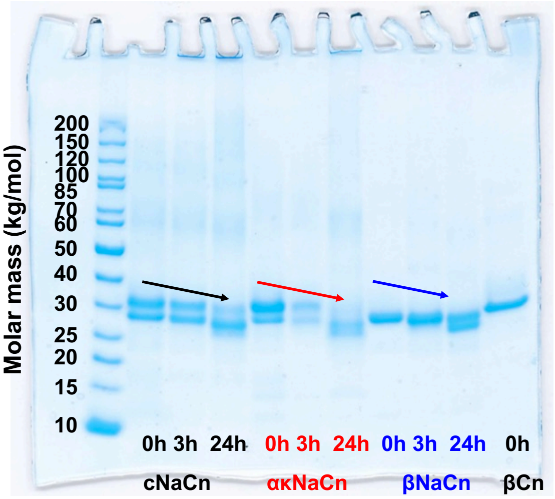

Figure S3. SDS-PAGE analysis reveals a reduction of c-, β- and ακNaCn monomer bands without cross-linking (0 h lanes) and appearance of compact c-, β- and ακSCNP species (3 h, 24 h) following incubation with mTGase for 3 and 24 hours respectively consistent with SCNP formation.

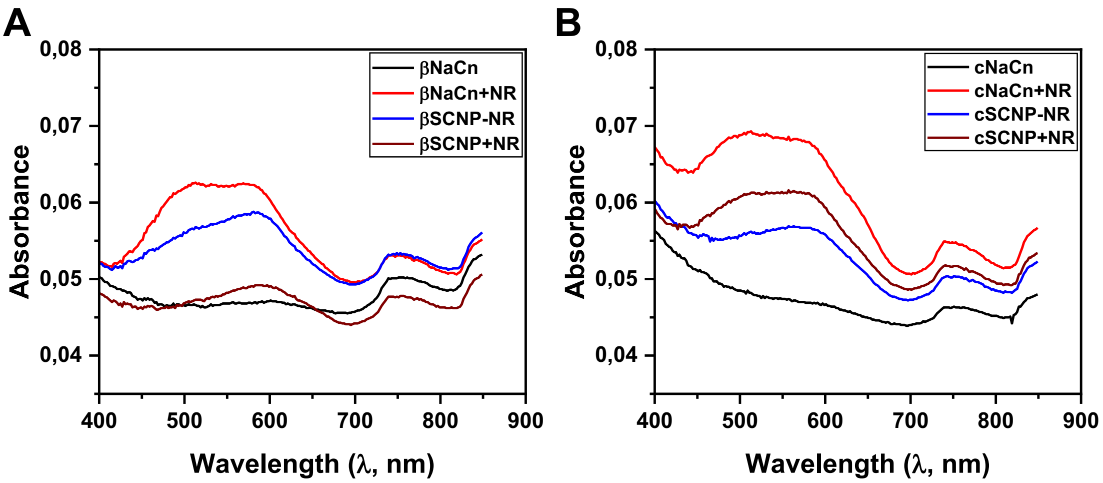

Figure S4. UV absorbance spectra of NR in (A) cNaCn and cSCNP samples, and (B) βNaCn and βSCNP samples. Absorption maxima are in the range 550-600 nm which was subsequently used as the excitation wavelength in fluorescence studies.

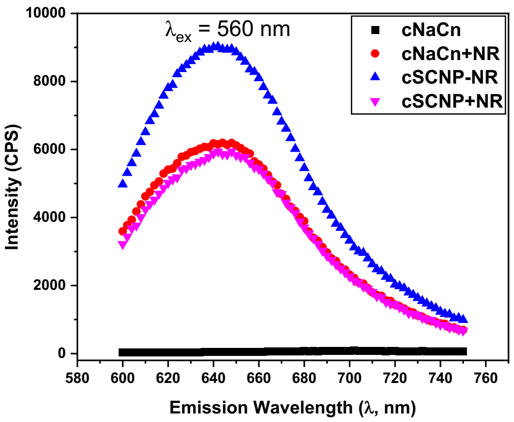

Figure S5. Batch-mode fluorescence spectra of NR encapsulated in cNaCn and cSCNP. High emission intensity of cSCNP-NR indicates higher sequestration of NR when cNaCn is cross-linked in the presence of NR, compared to cNaCn+NR and cSCNP+NR where NR was dispersed without and after cross-linking respectively. Notably, cSCNP-NR exhibits ca. twice the intensity of βSCNP-NR (Figure 4A) under the same conditions.

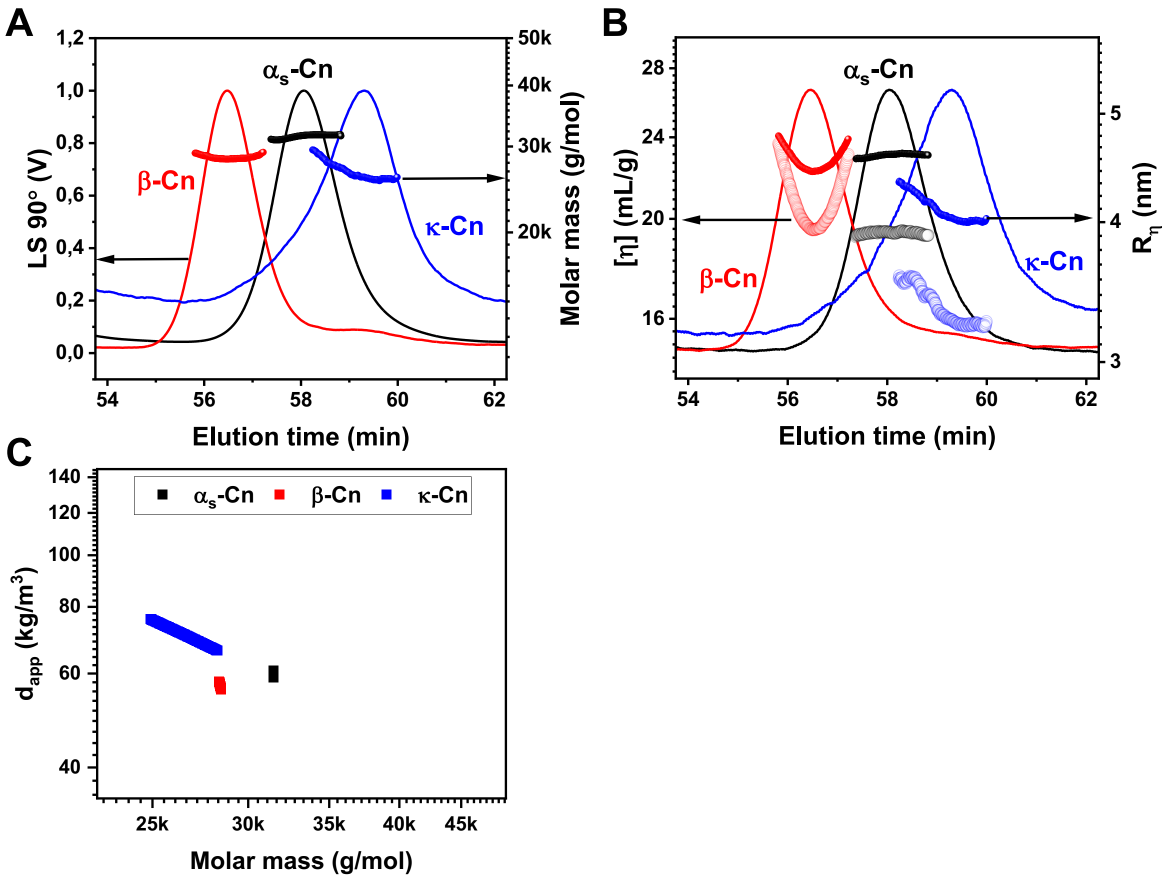

Figure S6. SEC-D5 results of (A) the chromatograms of commercial α_s-, β-, and κ-casein standards showing MM 25-30 kg/mol in congruence with the theoretical MM of casein, (B) the [η] and R_η properties of the same commercial α_s-, β-, and κ-casein standards, and (C) the apparent density profiles of the same commercial casein standards. The properties of these casein standards supports the observation of the monomeric state of cNaCn, βNaCn and ακNaCn before cross-linking shown in SDS-PAGE and SEC-D5 measurements.

Table S3. Key parameters of the commercial purified α_s-, β-, and κ-casein standards determined via SEC-D5 considering only the monomer peak of interest.

Sample	M_w (kg/mol)	Đ (M_w/M_n)	$R_{\eta,w}$ (nm)	$[\eta]_w$ (mL/g)
α_s-Cn	28.15	1.00	4.59	21.69
β-Cn	28.44	1.00	4.51	20.38
κ-Cn	26.48	1.00	4.09	16.32
cNaCn	26.12	1.00	4.37	20.13
βNaCn	27.18	1.00	4.75	24.96

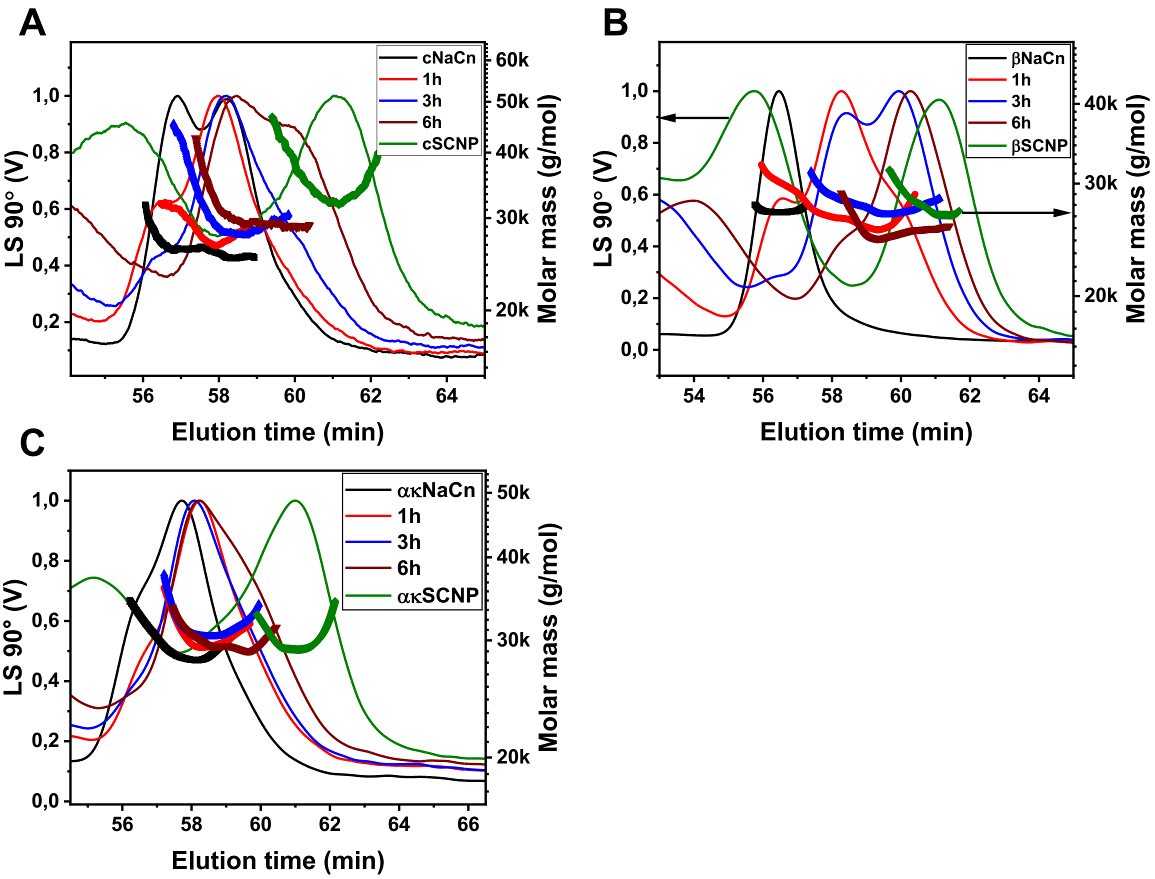

Figure S7. SEC-D5 chromatograms of (A) cNaCn, (B) βNaCn and (C) αĸNaCn, showing the relatively unchanging MM distribution in the ~ 30 kg/mol range during the development of the respective c-, β- and αĸSCNPs through 1, 3 and 6 hours, so that the final SCNP samples were collected after 24hours. The chromatograms shift towards later elution times on the right with increasing incubation time and the MM agrees closely with that of the commercial casein standards in Figure S6 and Table S2, and also with the c-, β- and αĸNaCn data in Figure 1B, Figure 2A and Table 1 in the main manuscript, This confirms the monomeric state of the c-, β- and αĸNaCn before cross-linking, and serves as a canonical signature of c-, β- and αĸSCNP formation after incubation with mTGase for 24 hours.

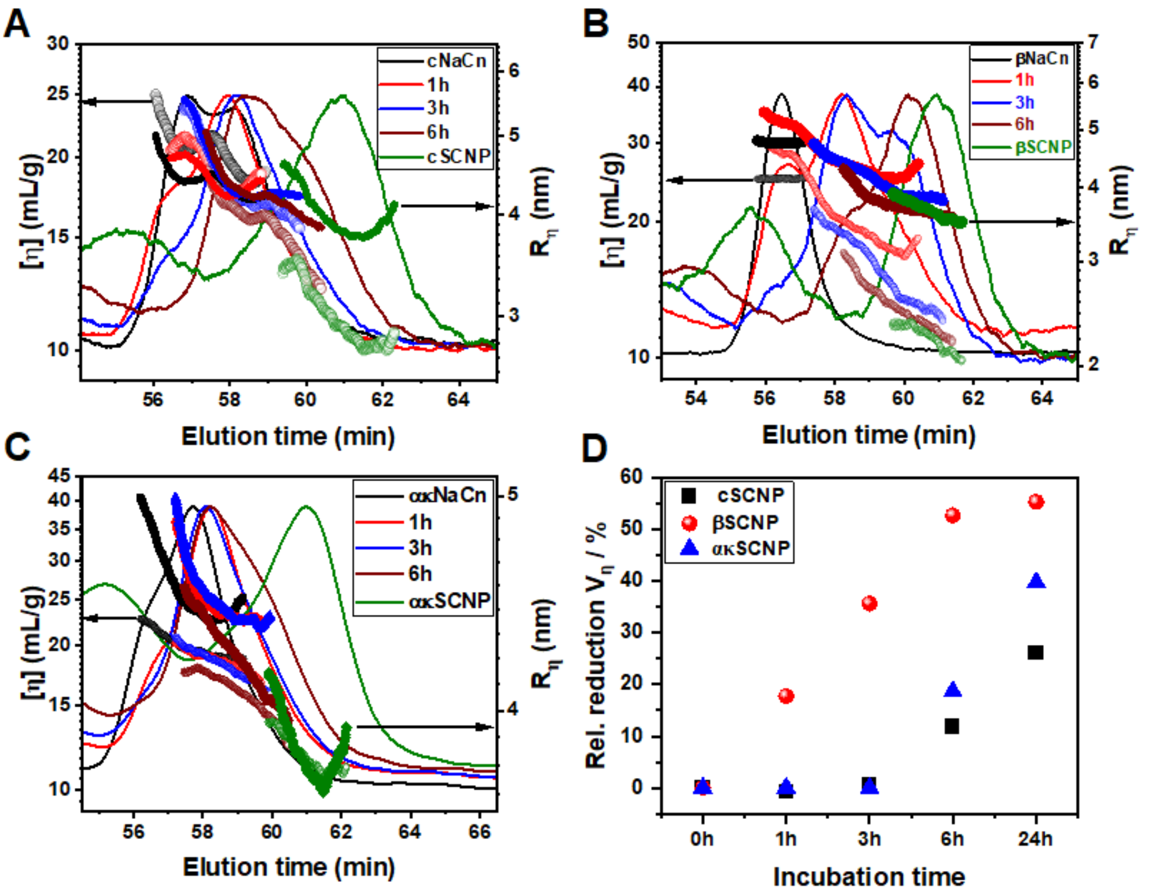

Figure S8. SEC-D5 chromatograms showing a general decrease in the R_η and [η] profiles of (A) cNaCn, (B) βNaCn, and (C) ακNaCn and their respective SCNPs after 1, 3 and 6 hours, with the final c-, β- and ακSCNP having been incubated with mTGase for 24 hours. In (D) the reduction in particle volume after internal cross-linking plotted for the stages of SCNP formation relative to th uncross-linked cNaCn, βNaCn and ακNaCn by assuming a global spherical conformation. This indicates compaction of the initial flexible chains into more compact structures.

Table S4. Key parameters of the cNaCn / cSCNP, βNaCn / βSCNP and ακNaCn / ακSCNP sample sets at different incubation times determined via SEC-D5.

Sample	M_w (kg/mol)	Đ (M_w/M_n)	$R_{\eta,w}$ (nm)	$[\eta]_w$ (mL/g)
cNaCn	26.1	1.0	4.4	20.13
1h	28.5	1.0	4.4	18.79
3h	30.4	1.0	4.4	17.64
6h	30.3	1.0	4.2	15.44
cSCNP	35.3	1.0	4.0	11.11

Sample	M_w (kg/mol)	Đ (M_w/M_n)	$R_{\eta,w}$ (nm)	$[\eta]_w$ (mL/g)
βNaCn	27.2	1.0	4.8	24.96
1h	27.1	1.0	4.5	20.7
3h	27.8	1.0	4.1	15.79
6h	25.4	1.0	3.7	12.63
βSCNP	27.8	1.	3.6	10.94

Sample	M_w (kg/mol)	Đ (M_w/M_n)	$R_{\eta,w}$ (nm)	$[\eta]_w$ (mL/g)
ακNaCn	29.5	1.0	4.5	19.92
1h	30.5	1.0	4.5	18.61
3h	31.5	1.0	4.5	18.33
6h	30.0	1.0	4.2	16.13
ακSCNP	30.1	1.0	3.8	11.94

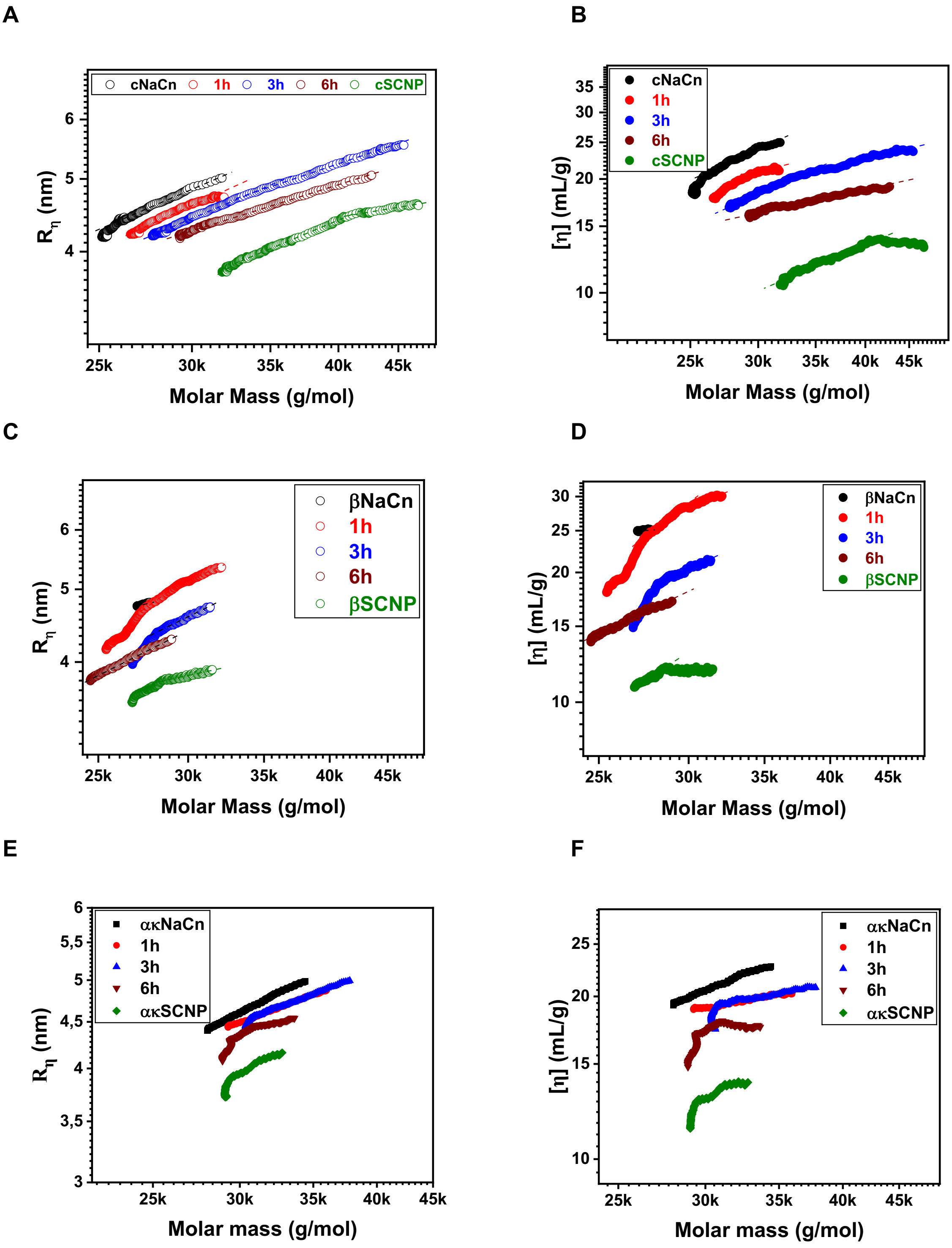

Figure S9. Scaling plots the dependence of R_η on MM to extract ν which bears conformation information (eq. S9) of (A) cNaCn, (C) βNaCn, and (E) ακNaCn and their respective SCNPs after 1, 3 and 6 hours, with the final c-, β- and ακSCNP having been incubated with mTGase for 24 hours. KHMS plots using the dependence of [η] on MM to extract the exponent α which bears conformation information (eq. S10) of (B) cNaCn, (D) βNaCn, and (F) ακNaCn and their respective SCNPs after 1, 3 and 6 hours, with the final c-, β- and ακSCNP having been incubated with mTGase for 24 hours. The narrow MM distribution of the casein derivates does not allow for the reliable determination of ν and α. However, the general trend in the reduction of R_η and [η] with increasing incubation time 0-24 h with mTGase is a sufficient indicator of global compaction during intramolecular cross-linking-driven SCNP formation.

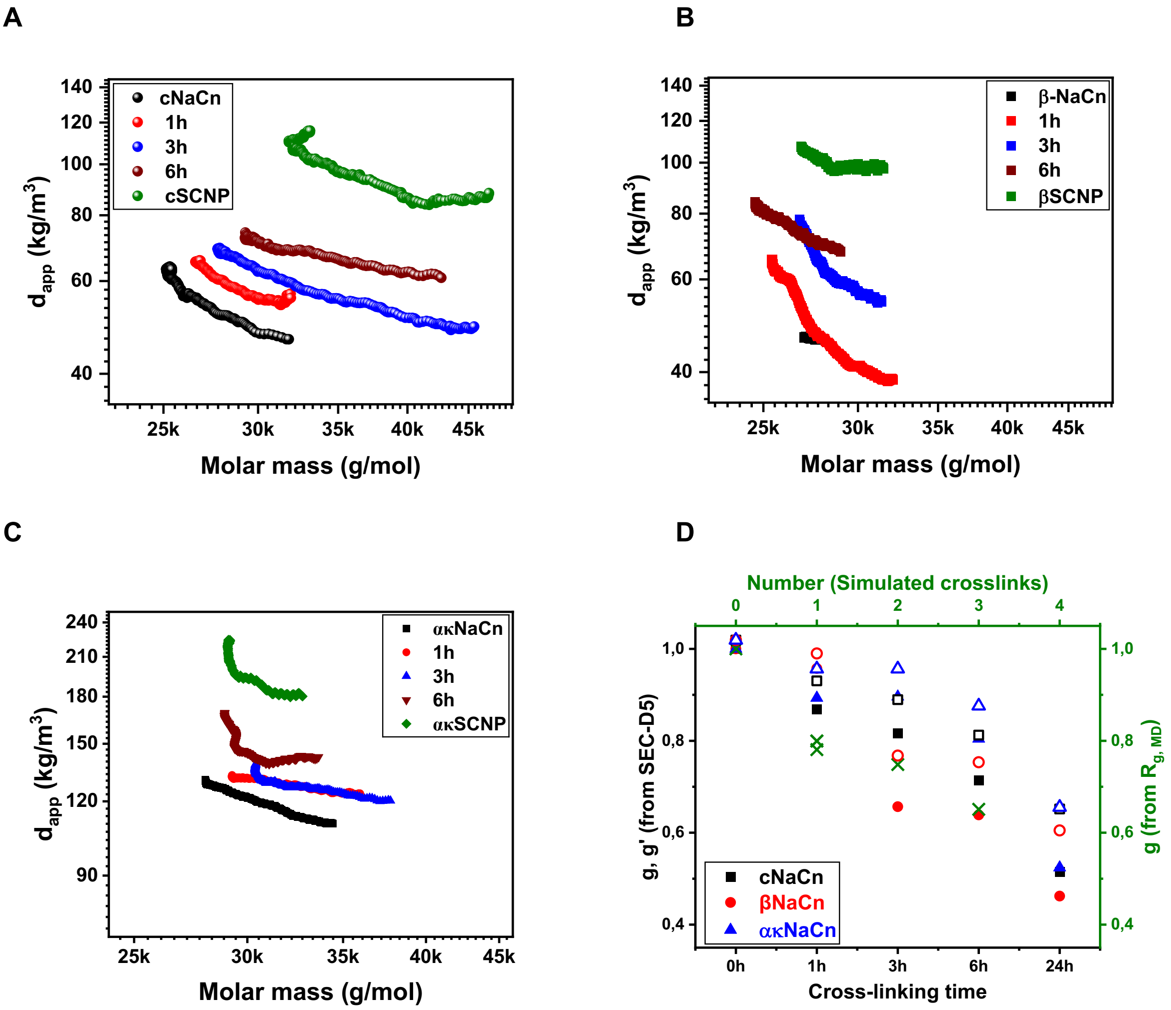

Figure S10. Apparent density (d_{app}) profiles of (A) cNaCn, (B) βNaCn, and (C) ακNaCn and their respective SCNPs after 1, 3 and 6 hours, with the final c-, β- and ακSCNP having been incubated with mTGase for 24 hours, calculated according to (eq. S11). The d_{app} increase for each sample set with increasing cross-linking time. Relatively constant molar masses (Figure S7) coupled with decreasing R_η (Figure S8) result in higher mass per unit volume, thus increasing d_{app} of the SCNPs. In (D) g (eq. S12, open symbols) and g' (eq. S13, closed symbols) from SEC-D5 data are calculated for cNaCn, βNaCn, and ακNaCn and their respective SCNPs after 1, 3 and 6 hours, and the final c-, β- and ακSCNP after 24 hours. The values are superimposed with the g (crossed symbols) from $R_{g,\,MD}$ of MD simulations of data described in Table S7 and Figure S17. By correlating the degree of contraction observed from the two approaches, we can estimate that each βSCNP is formed with maximum 3-4 mTGase induced isopeptide bonds. This is possible due to the fact that contraction factors as a measure, are independent from chemical structure, chain architecture and solvent conditions.

Table S5. Report of quality data and Xray scattering length densities used in the evaluation of the SEC-SAXS data.

Sample	ATSAS total quality estimate	X-ray SLD, 10^{-6} ($Å^{-2}$)
βNaCn	0.84	10.061
βSCNP	0.87	10.061
βSCNP-NR	0.85	10.061*

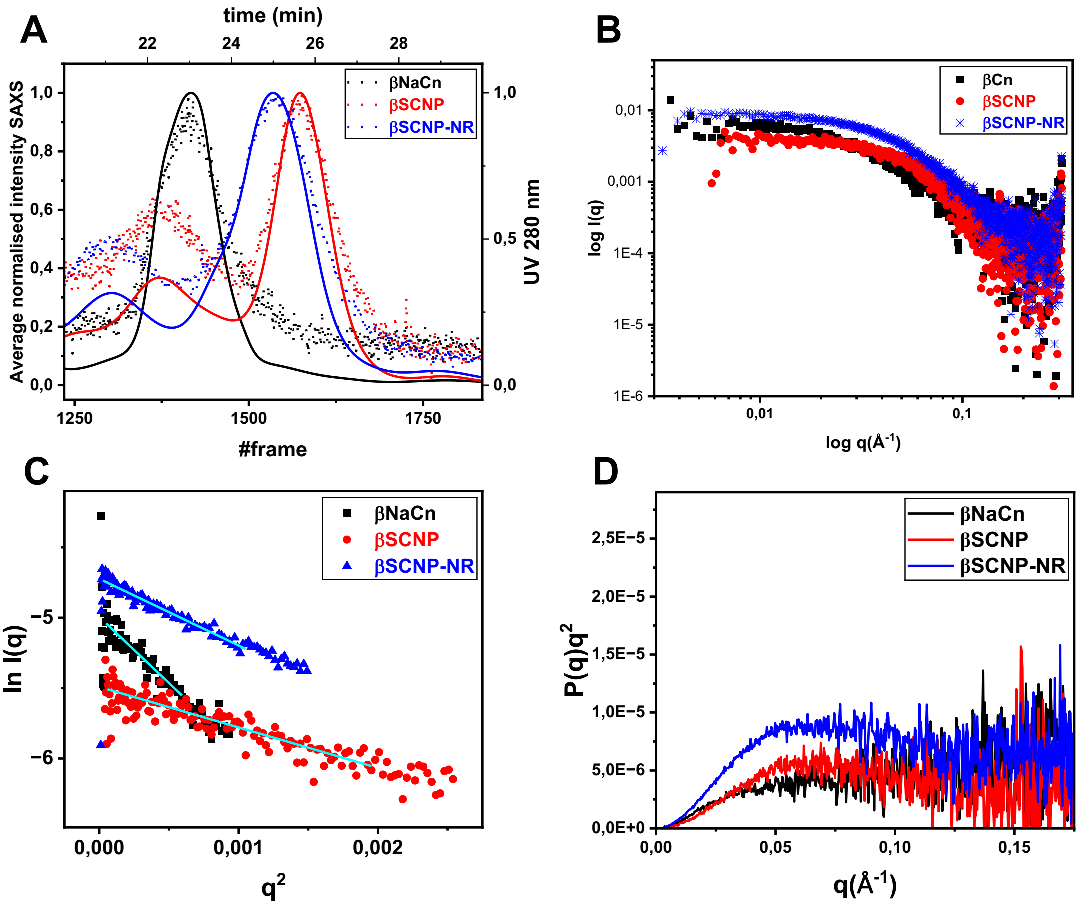

Figure S11. (A) SEC-SAXS chromatogram of βNaCn, βSCNP and βSCNP-NR showing the overlay of the UV and SAXS scattering intensity signals overlaid. (B) Original SAXS scattering profiles from the peak maxima of the monomer peak of each sample. (C) Plots of Guinier analyses of the SEC-SAXS data from which the R_g of βNaCn, βSCNP and βSCNP-NR in Table 1 of the main text were derived. (D) Original Kratky plots of βNaCn, βSCNP and βSCNP-NR showing overlapping data points in the inherently noisy high-q range.

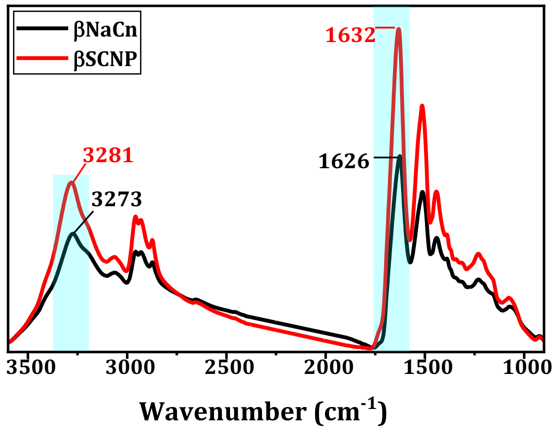

Figure S12. FTIR spectra of βNaCn overlaid with βSCNP to compare the effect of cross-linking by mTGase on the functional groups of the proteins. Small amide I band shift from 1626 to 1632 cm^{-1} and NH-stretching from 3273 to 3281 cm^{-1} after cross-linking is likely due to weaker hydrogen bonding and structural rigidity in the protein molecule.

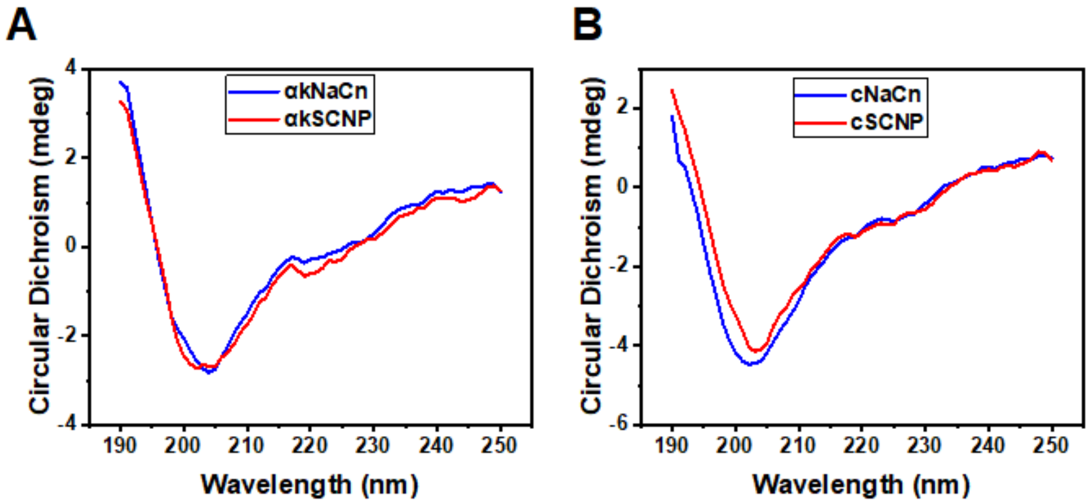

Figure S13. Circular dichroism shows no emergence of new secondary structure upon compaction of ακNaCn and cNaCn into ακSCNP and cSCNP respectively.

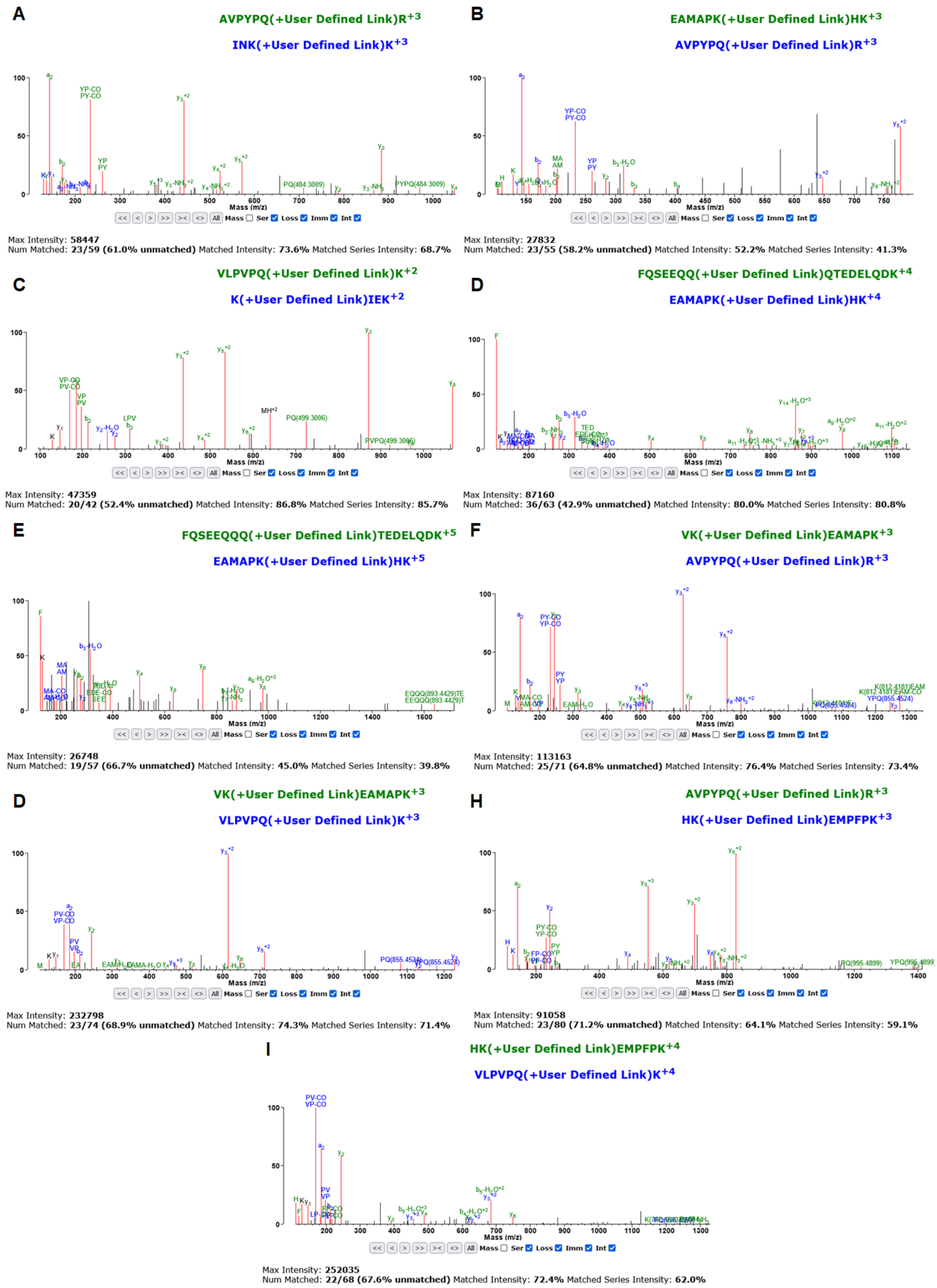

Figure S14. MS/MS spectra in A-I showing the assignment of mTGase induced intramolecular isopeptide cross-links in βSCNP from the software Protein Prospector. The Lys and Gln residues participating in the formation of K-Q cross-links are annotated above the spectrum, listed in Table S6, and visualised in Figure S15B.

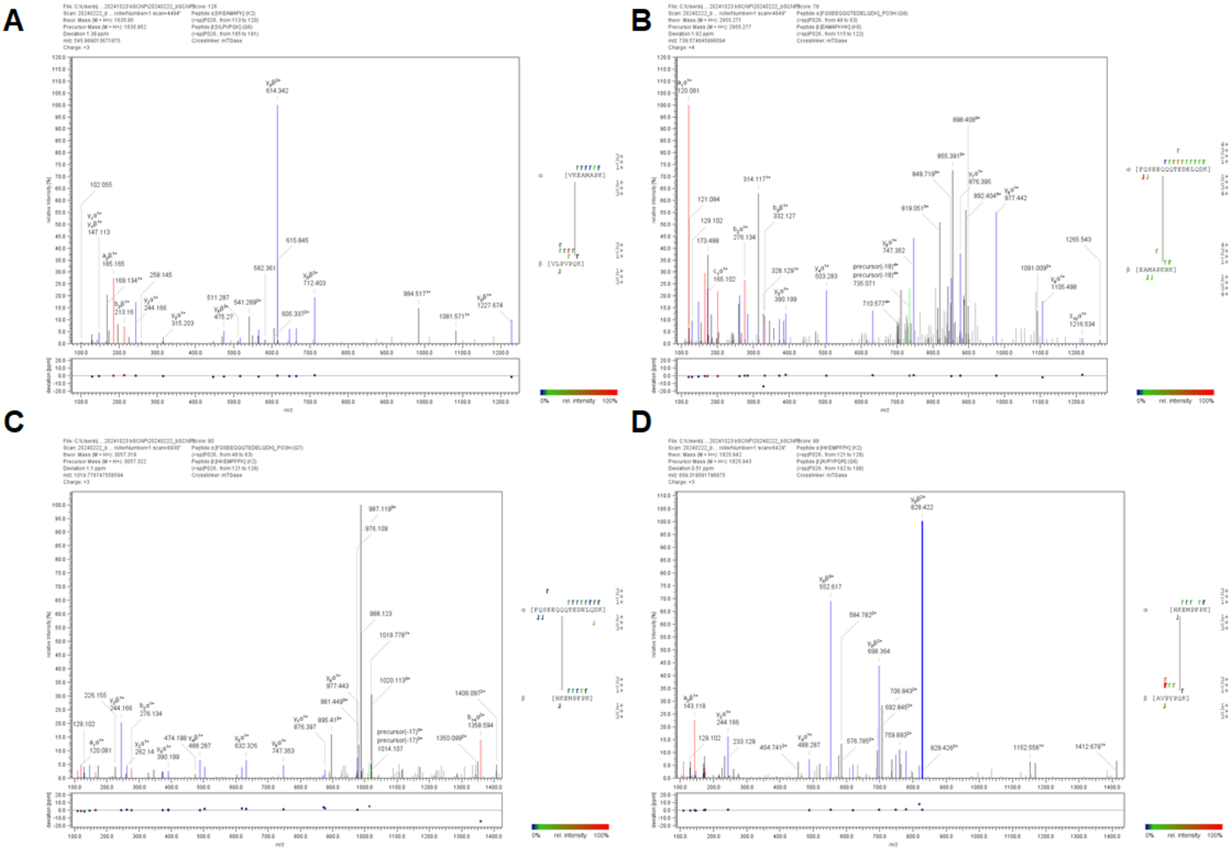
A
B
C
D

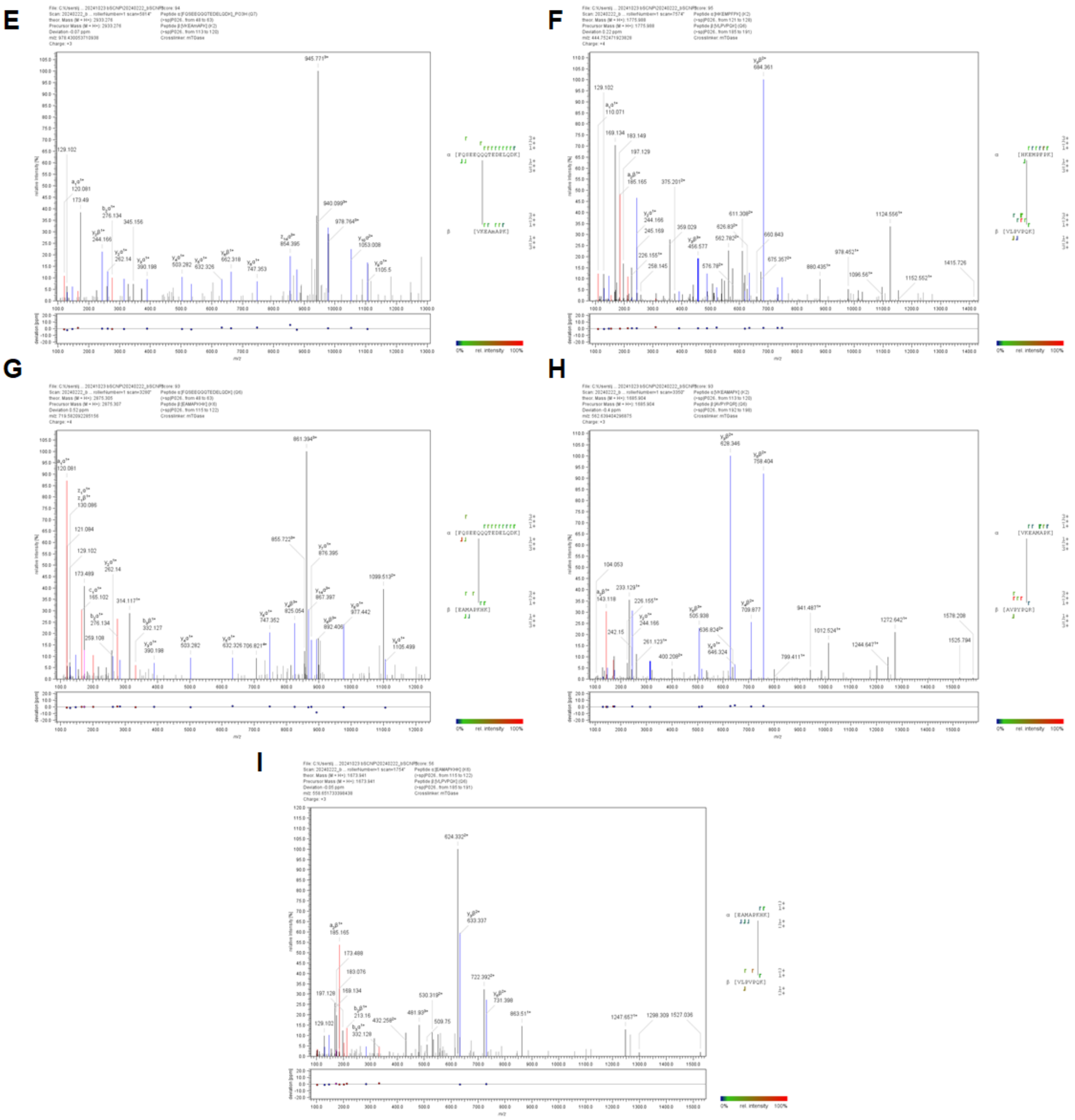

Figure S15. MS/MS spectra in A-I showing the assignment of mTGase induced intramolecular isopeptide cross-links in βSCNP from the software MeroX/StavroX. The Lys and Gln residues participating in the formation of K-Q cross-links are annotated on the righthand side of the spectrum, listed in Table S6, and visualised in Figure S15B.

Table S6. A list of the isopeptide cross-link hotspots identified in βSCNP near the N-terminal (24-55), midchain (98-122) and near the C-terminal (190-197) out of the 224 amino acid residues of the primary structure of β-casein.

m/z	z	ppm	Crosslinked Peptide[a]	RT[b]	MSMS Info	Score Difference[c]	XLink AA[d]
665.3882	2	12	AVPYP**Q**R	17.728	914	17.1	197
			**K**IEK				44
438.9274	3	11	AVPYP**Q**R	17.7353	918	5.3	197
			IN**K**K				43
575.3032	3	0.12	EAMAP**K**HK	18.574	1103	12.4	120
			AVPYP**Q**R				197
640.4037	2	1.3	VLPVP**Q**K	19.8132	1458	4.7	190
			**K**IEK				44
719.5821	4	0.53	FQSEEQ**Q**QTEDELQD K	24.4385	3280	12.7	54
			EAMAP**K**HK				120
575.8671	5	0.46	FQSEEQQ**Q**TEDELQD K	24.4515	3287	5.2	55
			EAMAP**K**HK				120
562.6394	3	-0.41	V**K**EAMAPK	24.592	3350	14.9	114
			AVPYP**Q**R				197
545.989	3	1.4	V**K**EAMAPK	27.1463	4494	18.7	114
			VLPVP**Q**K				190
609.3191	3	0.53	AVPYP**Q**R	31.43	6429	13.3	197
			H**K**EMPFPK				122
444.7525	4	0.28	H**K**EMPFPK	33.9883	7592	4.2	122
			VLPVP**Q**K				190

K and **Q** are the specific cross-link sites
[a] Active web links to the MS/MS spectrum of the cross-linked peptide on the Protein Prospector engine
[b] Retention time in the liquid chromatography separation
[c] Distinction between the top-ranked peptide spectral match and the next best alternative, greater difference ~ higher confidence
[d] Cross-link amino acid number in the canonical β-casein amino acid sequence

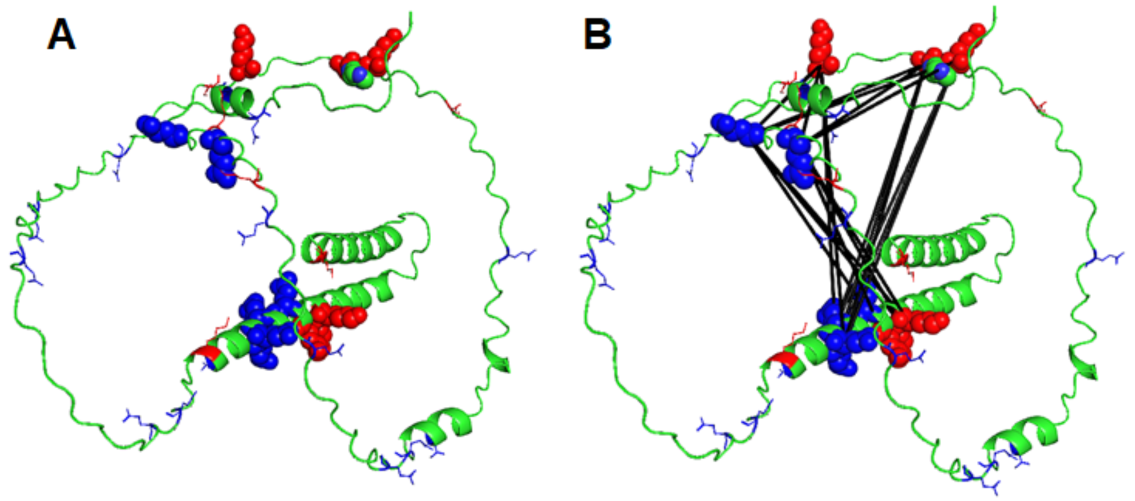

Figure S16: both structures in A and B show the Alphafold model (P02666) of a β-Cn molecule, with all the Lys and Gln residues in the β-Cn backbone marked in red and blue, respectively. Further, the residues which were identified to partake in mTGase catalysed isopeptide cross-linking are marked as spheres. The black lines in B show the hotspot network of isopeptide cross-links with residue numbers listed in Table S6.

Table S7. Table listing the system of three isopeptide cross-links identified by XL-MS which were applied as covalent constraints in the full atomistic MD simulation of β-Cn-βSCNP structure analysis.

System of modelled cross-links	$R_{g, MD}$ [nm]	Standard deviation [nm]	% of decrease as compared to pristine P02666 β-casein	Apparent Flory exponent (ν_{app})*; ±0.01 - 0.02 (fit error)
β-Cn (P02666)	3.78	-	0	0.708
K_{44}-Q_{197}	3.38	0.09	10.6	0.691
K_{120}-Q_{54}	3.34	0.50	11.6	0.689
K_{122}-Q_{190}	3.38	0.29	10.5	0.666
K_{120}-Q_{54}, K_{122}-Q_{190}	3.27	0.25	13.5	0.665
K_{44}-Q_{197}, K_{120}-Q_{54}, K_{122}-Q_{190}	3.05	0.15	19.3	0.654

* see Figures S17 and S18

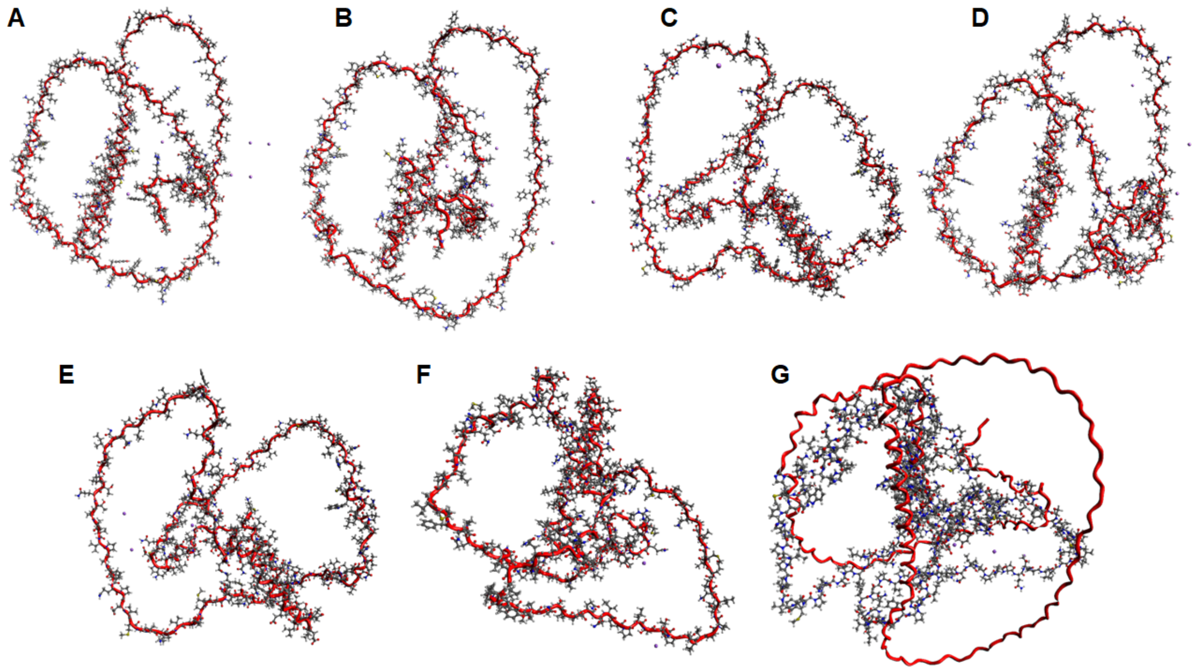

Figure S17. MD simulations of the conformations of (A) the pristine β-Cn molecule obtained from the Protein Databank file AF-P02666-F1-model_v4. XL-MS-constrained MD simulations of the physical impact of single cross-link on β-Cn (B) K_{44}-Q_{197}, (C) K_{120}-Q_{54}, (D) K_{122}-Q_{190}, (E) the physical impact of two cross-links K_{120}-Q_{54}+K_{122}-Q_{190} and (F) the physical impact of three cross-links K_{122}-$Q_{190}$$K_{44}$-$Q_{197}$+$K_{120}$-$Q_{54}$+$K_{122}$-$Q_{190}$ on the conformation of β-Cn. (G) Size comparison by the superimposition of the red ribbon of the pristine β-Cn ribbon from (A) mapped onto a conformation of intensely cross-linked β-Cn in (F), Images not to scale.

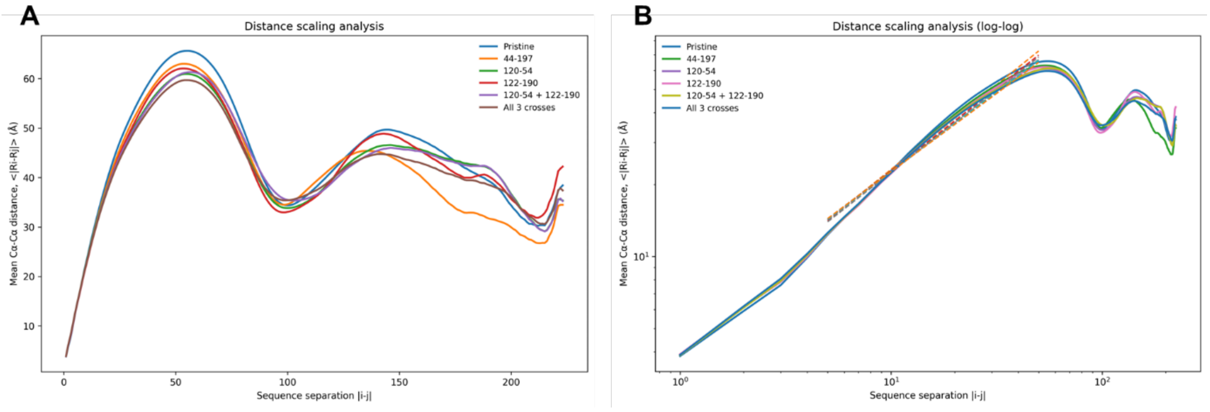

Figure S18. (A) The linear scaling representation shows a pronounced deviation from ideal power-law behavior, with the curves bending downward beyond sequence separations of approximately 50 residues. Notably, this effect is already observed for pristine β-Cn in the absence of cross-links, indicating that the system does not exhibit an extended asymptotic scaling regime and retains significant finite-size and correlation effects. (B) The double-log representation reveals an intermediate regime that is approximately linear, allowing for the estimation of an apparent Flory exponent. Within this regime, a slight decrease in the exponent is observed for the more extensively cross-linked βSCNP constructs, consistent with a modest increase in chain compaction (see Table S7).

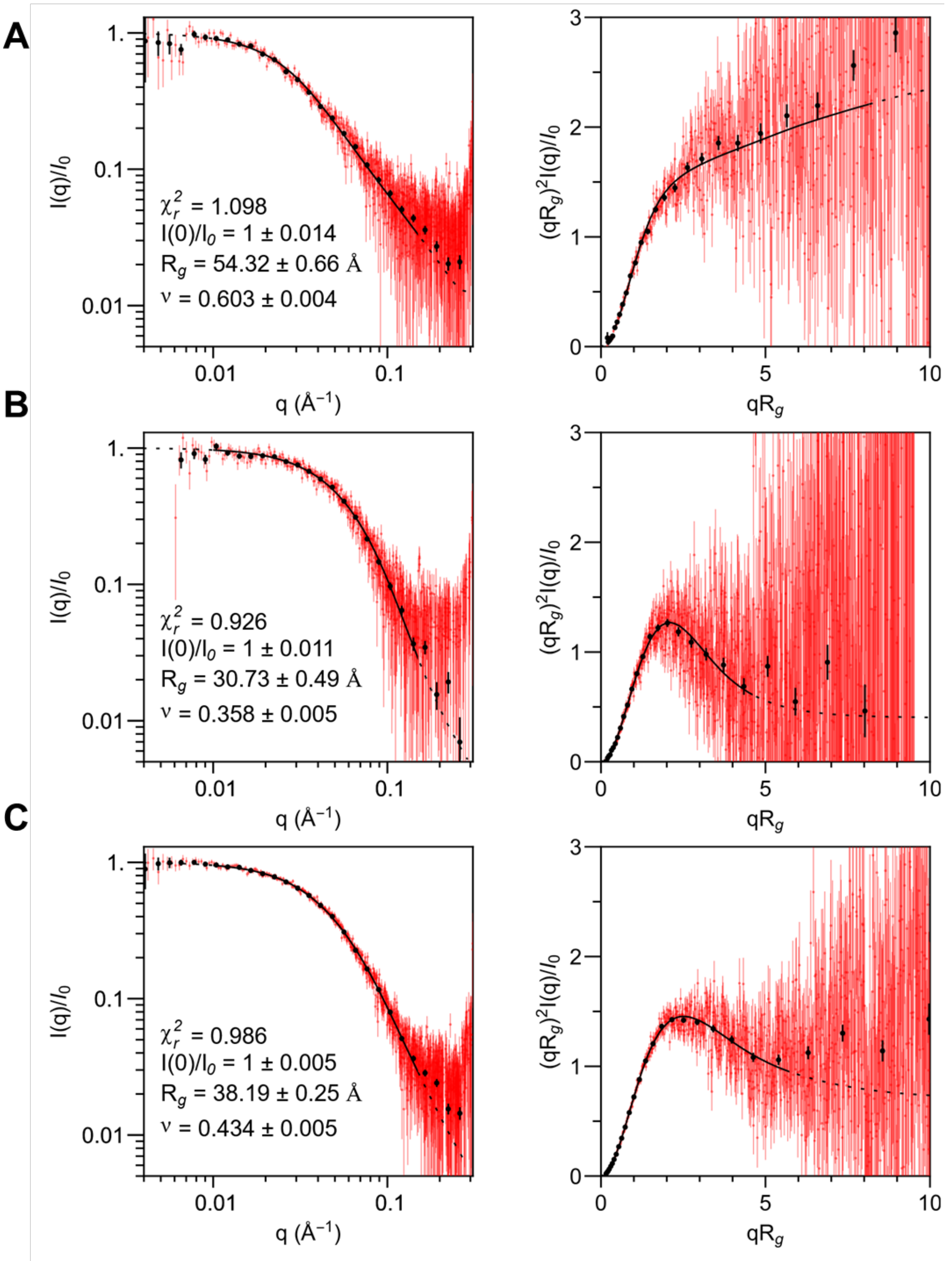

Figure S19. Log-log plot and dimensionless Kratky plots of SAXS scattering data and fit of (A) βNaCn, (B) βSCNP and (C) βSCNP-NR, analysed using molecular form factor (MFF) approach developed from simulations of disordered proteins available at http://sosnick.uchicago.edu/SAXSonIDPs.
